# Supplementary material for: Signatures of Edge States in Antiferromagnetic Van der Waals Josephson Junctions
Source: Adv Mater. 2026 Jan 30;38(51):e21250. doi: 10.1002/adma.202521250 (PMC13568857; doi:10.1002/adma.202521250)
Supplement: Supplementary file 1 [file ADMA-38-e21250-s001.pdf]

Supporting Information

Signatures of Edge States in Antiferromagnetic van der Waals Josephson Junctions

C. González-Sánchez ,[1,2,3] I. Sardinero ,[4,2] J. Cuadra,[1,2,3] A. Spuri ,[5] J. A. Moreno,[1,2] H. Suderow ,[1,2,3] E. Scheer ,[5] P. Burset ,[4,2,3] A. Di Bernardo ,[5] R. Seoane Souto ,[6,7] and E. J. H. Lee [1,2,3,7,*]

[1] *Departamento de Física de la Materia Condensada, Universidad Autónoma de Madrid, 28049 Madrid, Spain*

[2] *Condensed Matter Physics Center (IFIMAC), Universidad Autónoma de Madrid, 28049 Madrid, Spain*

[3] *Instituto Nicolás Cabrera, Universidad Autónoma de Madrid, 28049 Madrid, Spain*

[4] *Department of Theoretical Condensed Matter Physics, Universidad Autónoma de Madrid, 28049 Madrid, Spain*

[5] *Department of Physics, University of Konstanz, Universitätsstraße 10, 78464 Konstanz, Germany*

[6] *Instituto de Ciencia de Materiales de Madrid (ICMM), Consejo Superior de Investigaciones Científicas (CSIC), Sor Juana Inés de la Cruz 3, 28049 Madrid, Spain*

[7] *Laboratorio de Transporte Cuántico, Unidad Asociada UAM/ICMM-CSIC, Madrid, Spain*

[*] eduardo.lee@uam.es

CONTENTS

S1. Sample characterization S3

A. Thickness and structural characterization of $NiPS_3$ flakes S3

B. Electrical Characterization of few-layer $NbSe_2$ flakes S6

S2. Summary of samples S7

S3. Additional samples S9

A. Device B S9

B. Samples prepared in air S11

S4. Additional data: Device A S13

A. Device fabrication S13

B. Supercurrent interference in device A for different angle θ S13

C. Temperature dependence of the critical current S17

D. Magnetic field dependence of the critical current S21

1. Zero-width asymmetric SQUID model S23

2. Finite-width asymmetric SQUID model S24

3. Relative angle between localized channels S24

S5. Microscopic model S27

References S31

S1. SAMPLE CHARACTERIZATION

A. Thickness and structural characterization of $NiPS_3$ flakes

To characterize the thickness and homogeneity of $NiPS_3$ flakes, we employed atomic force microscopy (AFM) and Raman spectroscopy on crystals directly exfoliated onto Si/SiO_x substrates (Figs. S1 and S2). These complementary techniques allowed us to relate the thickness of a $NiPS_3$ flake to its optical contrast, which was ultimately used to estimate an upper bound of ≈ 10 nm to the thicknesses of the barriers in our devices.

Height profiles extracted from the AFM data are shown in Fig. S1c, which were taken in a region with few layers, corresponding to the orange line shown in the optical image (Fig. S1b).

Raman spectra were acquired to identify and confirm the layer-dependent vibrational modes of $NiPS_3$. Characteristic Raman peaks exhibited systematic shifts and intensity changes as a function of layer number (Fig. S2c), providing a complementary and rapid method to distinguish mono-, bi-, and few-layer regions within the same flake [1, 2].

By combining the quantitative height information from AFM with the spectral fingerprints obtained from Raman spectroscopy, we successfully identified regions with different numbers of layers within individual $NiPS_3$ flakes. Additionally, both AFM and Raman measurements confirmed the high degree of homogeneity within each region of the flakes.

To assess the crystalline order in our $NiPS_3$ crystals, we have characterized flakes exfoliated in air onto Si/SiO_x substrates by scanning transmission electron microscopy (STEM) [3]. To this end, STEM lamellae were prepared with the aid of a focused ion beam system. Cross-sectional STEM micrographs (unpublished data, not shown), taken for different magnifications, demonstrate a high crystalline order of our samples. To ensure statistical relevance, similar measurements were performed on multiple flakes and across different regions. In all cases, the structural order is preserved over extended length scales, spanning at least several micrometers.

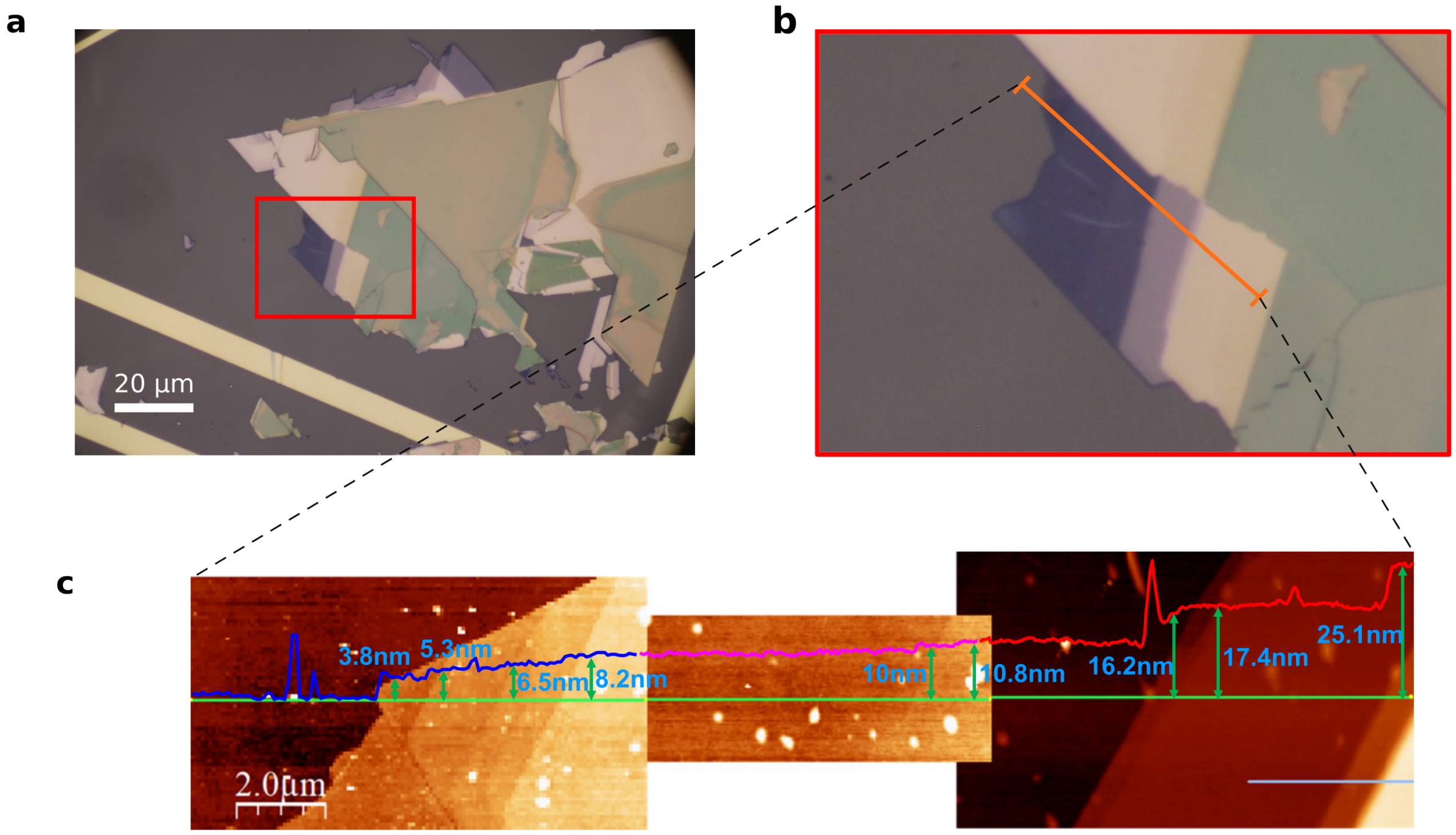

FIG. S1. **Thickness and homogeneity of NiPS$_3$.** **a,** Optical micrograph of a NiPS$_3$ flake exhibiting regions of varying thicknesses. **b**, Zoomed-in view, highlighting the specific area of interest. **c,** AFM topography of the area of interest, with the corresponding height profile. The part of the flake with purple contrast is still in the few-layer limit.

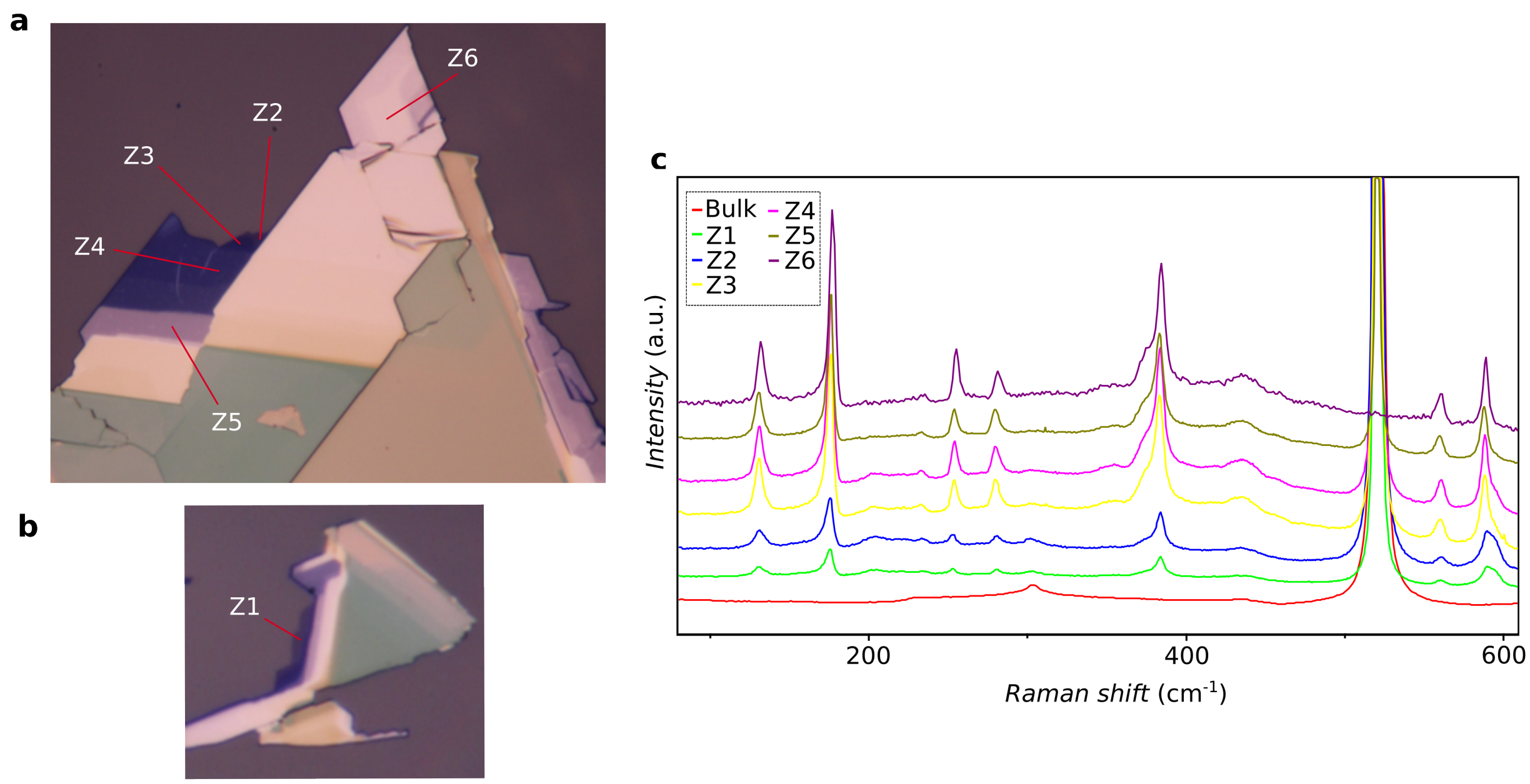

FIG. S2. **Raman spectroscopy of $NiPS_3$ flakes**. **a, b,** Optical micrograph of a $NiPS_3$ flake. The regions in which the spectra were acquired are labeled as Z1, Z2, etc. **c,** Raman spectra showing distinct vibrational modes with varying number of layers within the flake.

B. Electrical Characterization of few-layer $NbSe_2$ flakes

Fig. S3 displays a measurement of the differential conductance of a few-layer $NbSe_2$ flake, similar to those used as superconducting contacts in our antiferromagnetic junctions, as a function of the bias current and of the temperature. In this case, the critical current is approximately 300 μA at 2 K. Typical critical currents of similar flakes varied between 0.1-1 mA at low temperatures.

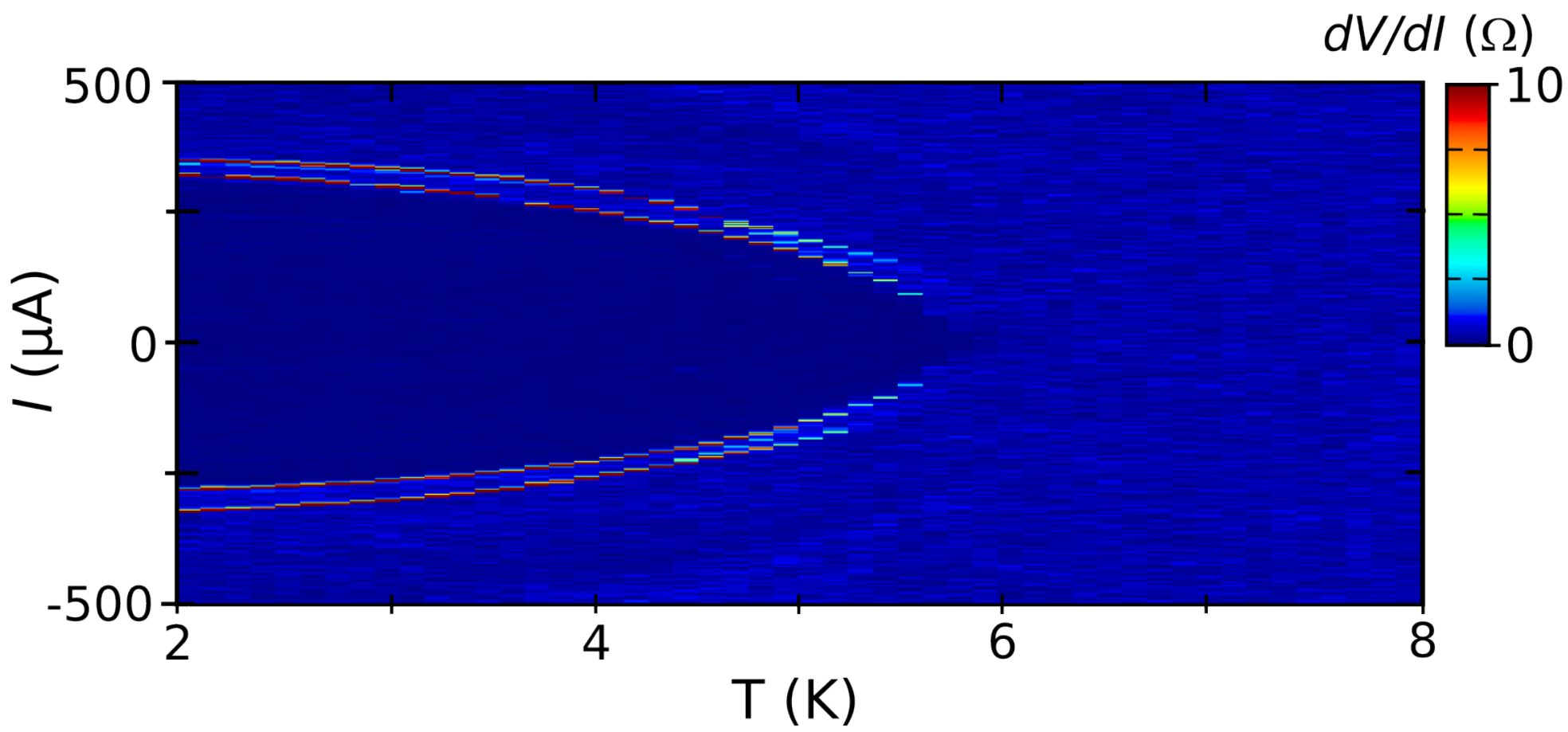

FIG. S3. Differential resistance, $dV(I)/dI$, of a few-layer $NbSe_2$ flake as a function of bias current and temperature.

S2. SUMMARY OF SAMPLES

TABLE S1: Summary of all samples investigated in this work. The last four entries, separated by the thick horizontal line, correspond to samples that showed device inhomogeneities, such as thickness variations or cracks in the flakes. For this reason, these samples were not studied in detail. Note that for the sample statistics, we have only considered samples that did not show clear inhomogeneities.

**Sample #**	**Transport characteristics**	**Observations**
**Device A (K06)**	Supercurrent detected, SQUID-like oscillations	Prepared in N_2 glovebox
**Device B (K11)**	Supercurrent detected, SQUID-like oscillations	Prepared in N_2 glovebox
X56 (Section S3.B)	Abrupt jumps of supercurrent with B	Prepared in air
X66 (Section S3.B)	Abrupt jumps of supercurrent with B	Prepared in air
X71 (Section S3.B)	Supercurrent gradually suppressed with no oscillations up to $B = 6$ T	Prepared in air
K01	One of the $NbSe_2$ flakes showed a finite resistance	Prepared in N_2 glovebox
K02	No dissipationless current, zero-bias dV/dI peak	Prepared in N_2 glovebox
K03	Zero-bias dV/dI peak, high resistance junction ($R_N \approx 4$ kΩ)	Prepared in N_2 glovebox

Continued on next page

**Sample #**	**Transport characteristics**	**Observations**
K07	Zero-bias dV/dI peak, high resistance junction ($R_N \approx 2$ kΩ)	Prepared in N_2 glovebox
K09	One of the $NbSe_2$ flakes showed a finite resistance	Prepared in N_2 glovebox
K04	Supercurrent detected. Consistent with a single localized channel with width ≈ 80 nm	Prepared in N_2 glovebox. $NbSe_2$ flakes with thickness variations
X45	Supercurrent detected, SQUID oscillations	Prepared in air. $NbSe_2$ with crack
X61	Zero-bias dV/dI peak, high resistance junction ($R_N \approx 0.6$ kΩ)	Prepared in air. $NiPS_3$ with wrinkles
X62	Zero-bias dV/dI peak, high resistance junction ($R_N \approx 0.6$ kΩ)	Prepared in air. $NiPS_3$ with wrinkles

S3. ADDITIONAL SAMPLES

A. Device B

In this section, we present data from device B, a second device that showed Josephson coupling and that was prepared using the same procedure as that used for device A in the main text. Fig. S4a shows an optical micrograph of the device, where the limits of the few-layer $NbSe_2$ and $NiPS_3$ flakes are highlighted by black and magenta dashed lines, respectively.

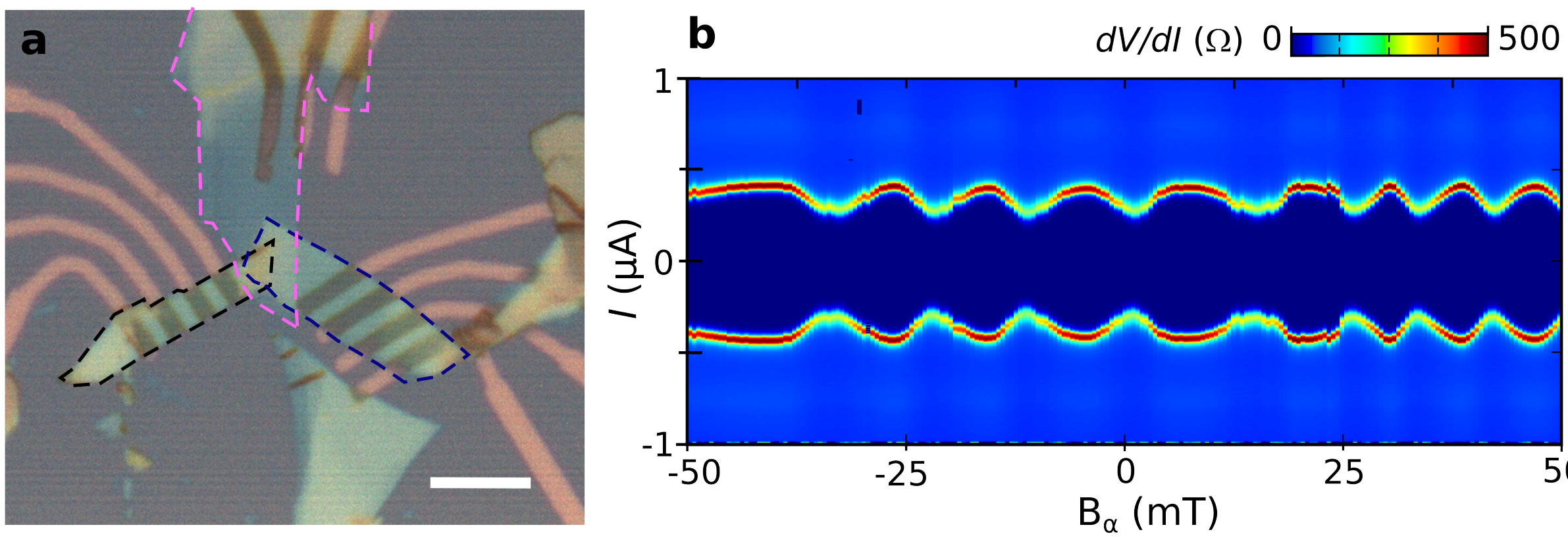

FIG. S4. (a) Optical micrograph of device B. The dark (magenta) dahsed lines denote the limits of the few-layer $NbSe_2$ ($NiPS_3$) flakes. The scale bar corresponds to 10 μm. (b) $dV(I)/dI$ as a function of the external magnetic field, B, applied along the ab-plane of the $NiPS_3$ barrier.

Electrical transport measurements were taken down to 250 mK using a ^{3}He insert and a solenoid magnet from Oxford Instruments. As for device A, the magnetic field, B, is applied along the ab-plane of the $NiPS_3$. However, in this setup, the direction of the magnetic field is fixed and thus, we were unable to study the angle dependence of the supercurrent interference. Fig. S4b shows a measurement of dV/dI as a function of the applied bias current and of B. Similar to device A, we observe SQUID-like oscillations of the supercurrent with magnetic field, instead of a Fraunhofer pattern. For this device, the maxima of the supercurrent is $I_s \approx 500$ nA, the normal resistance is $R_n \approx 60\ \Omega$, and the junction area, $A \approx 14.5\ \mu\text{m}^2$. From these values, we obtain $R_nA \approx 870\ \Omega\mu\text{m}^2$, $J_s \approx 3.45$ A/cm^2 and $IsR_n \approx 30\ \mu$eV, all comparable to device A. Moreover, the average period of SQUID-like

oscillations is $\overline{\Delta B} \approx 12.5$ mT, from which we estimate the area of the junction perpendicular to the field, $S \approx 1.65 \times 10^{-1} \mu\text{m}^2$, and the separation between localized channels, $W \approx 8.27$ μm, consistent with the sample dimensions (note that for the latter, $d + 2\lambda_L$ was taken to be 20 nm as in the main text). Finally, Fig. S5 displays a $dV(I)/dI$ measurement taken up to 2 T. For such high magnetic fields, the oscillations persist but are less regular than those shown in Fig. 4 in the main text, which in that case were taken for $B \parallel \hat{y}$ ($\theta = 90°$). Instead, the oscillations in Fig. S5 resemble those observed in device A for measurements taken for different angles (see section S4 B).

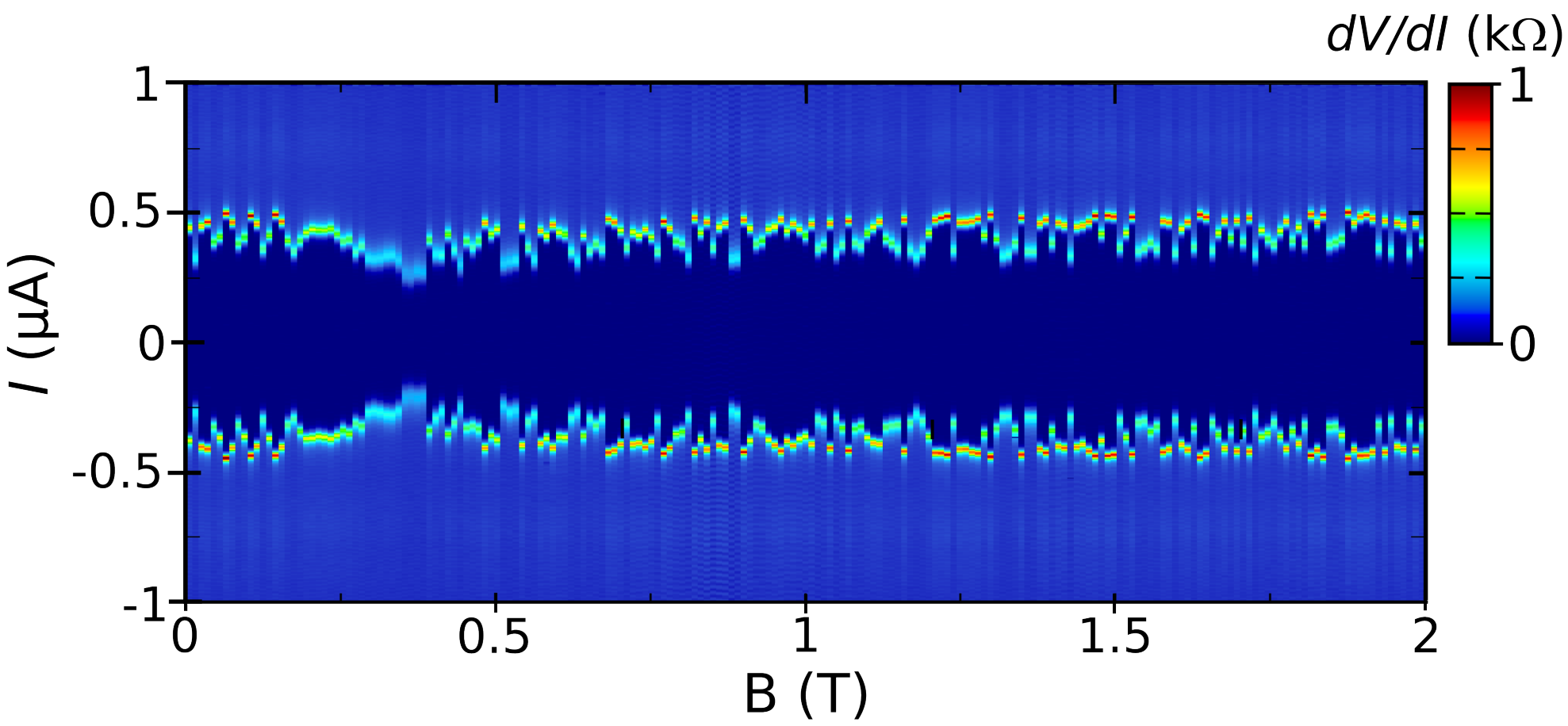

FIG. S5. $dV(I)/dI$ as a function of the external magnetic field, B, applied along the *ab*-plane of the $NiPS_3$ barrier at higher magnetic fields.

B. Samples prepared in air

Here, we present the electronic transport data of the three samples prepared in air that displayed Josephson coupling. Fig. S6 shows the $dV(I)/dI$ measurements taken as a function of the applied magnetic field for samples (a) X56, (b) X66 and (c) X71. Clearly, none of the samples display a Fraunhofer pattern behavior. Samples X56 and X66 show abrupt variations of the supercurrent as the magnetic field is swept. We hypothesize that these jumps are related to the oxidation of the antiferromagnetic barrier. Indeed, for sample X71, a fresh crystal of $NiPS_3$ was employed for exfoliating the few-layer barrier and the abrupt jumps are absent. For this sample, we observe that I_s is gradually suppressed by the magnetic field, but no oscillations are observed. We attribute this behavior to the formation of a single localized channel in the vdW Josephson junction (i.e., $n = 1$).

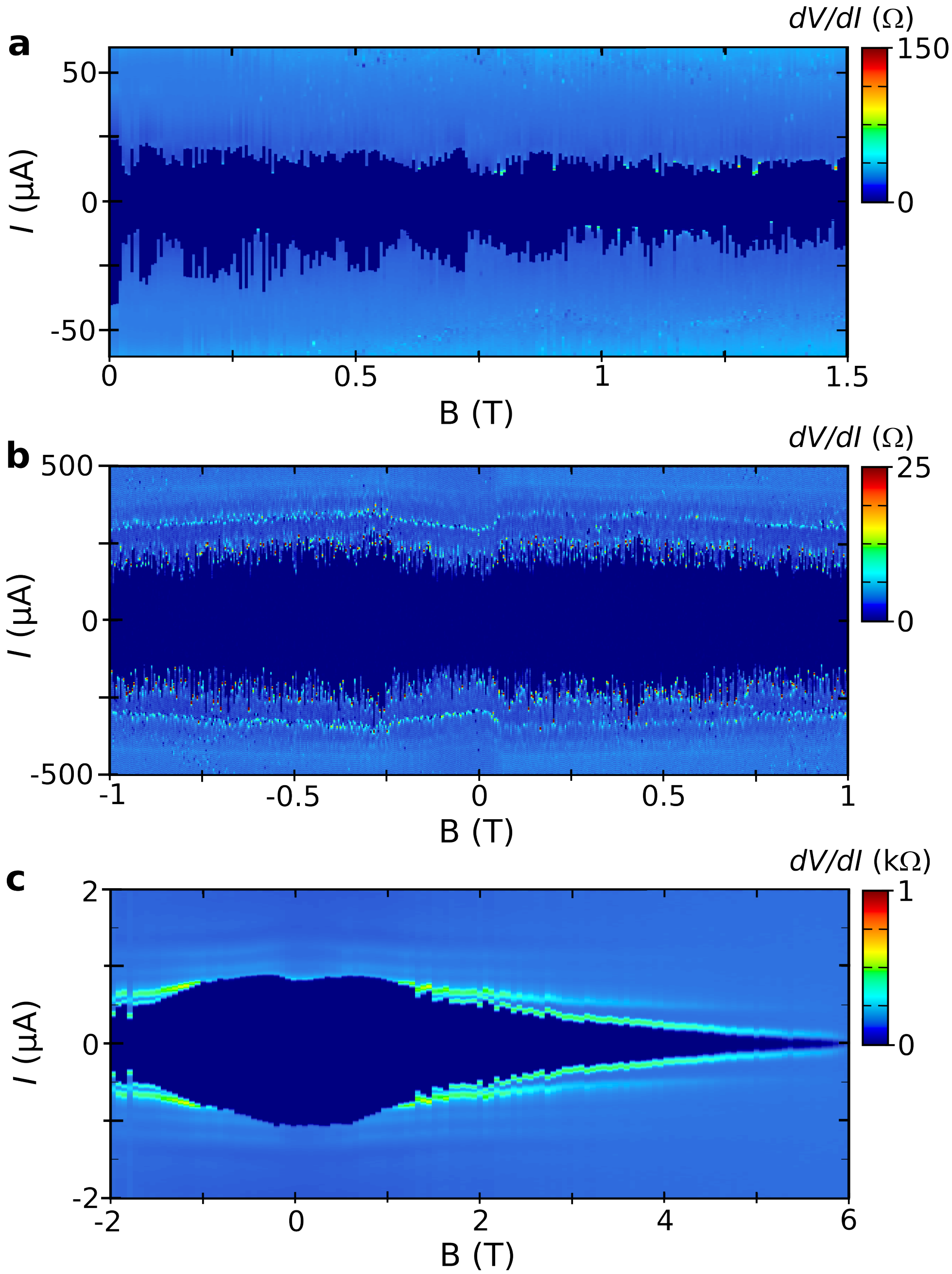

FIG. S6. $dV(I)/dI$ as a function of an external magnetic field applied along the *ab*-plane of the $NiPS_3$ barrier for samples (a) X56, (b) X66, and (c) X71.

S4. ADDITIONAL DATA: DEVICE A

A. Device fabrication

Figure S7 illustrates the sequence of steps involved in assembling the $NbSe_2/NiPS_3/NbSe_2$ heterostructure described in the Methods section of the main text. The process begins with the transfer of a bottom $NbSe_2$ flake onto pre-patterned Cr/Au contacts. Subsequently, a thin $NiPS_3$ flake is aligned with the bottom superconducting flake using a PDMS stamp under an optical microscope and then transferred to form the intermediate $NiPS_3/NbSe_2$ stack. Finally, the top $NbSe_2$ flake is transferred to complete the vertical junction. After the stacking sequence, the device is encapsulated with an hBN flake to ensure environmental stability and to preserve the cleanliness of the interfaces. These steps are representative of the standard dry-transfer procedure employed for all devices discussed in this work.

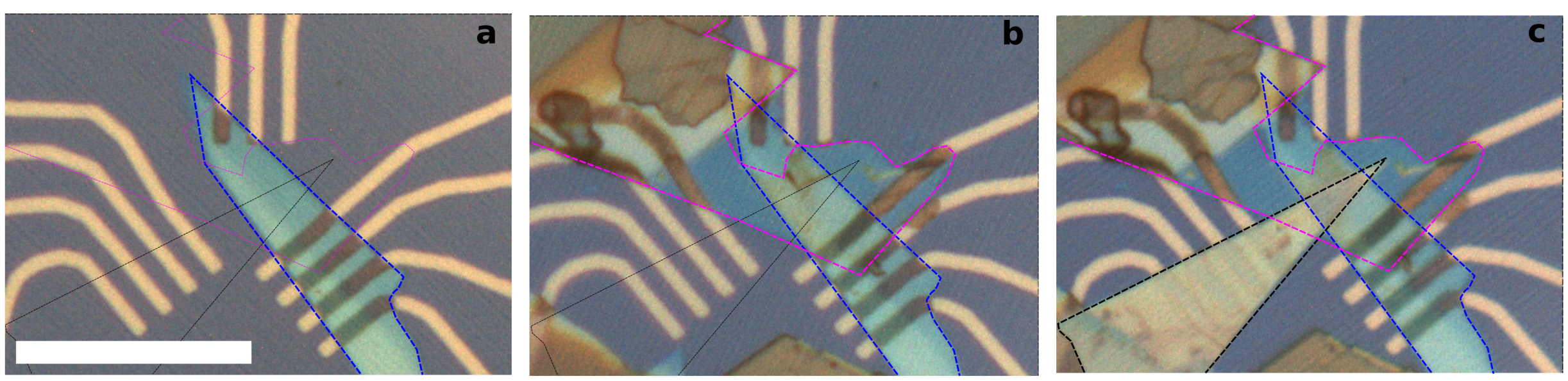

FIG. S7. Fabrication steps of the van der Waals heterostructure.: (a) Transfer of the bottom $NbSe_2$ flake onto pre-patterned Cr/Au electrodes; (b) Assembly of the intermediate $NiPS_3/NbSe_2$ stack, and (c) final $NbSe_2$/ $NiPS_3$/ $NbSe_2$ heterostructure obtained after transferring the top $NbSe_2$ layer. The completed device is subsequently encapsulated with an hBN flake (not shown here). Scale bar is 30 μm.

B. Supercurrent interference in device A for different angle θ

In the main text, we have shown $dV(I)/dI$ as a function of the applied magnetic field for $\theta = -30°$ and 65° up to $B = 1$ T (Fig. 2) and $\theta = 90°$ up to $B = 6$ T (Fig. 4). Here, we present measurements taken for $\theta =$ (a) 90°, (b) 130°, and (c) −60° up to $B = 1$ T. Note that the measurement shown in Fig. S8a was employed for obtaining the supercurrent

spatial density profile displayed in Fig. 2e in the main text. By contrast, Figs. S8b and S8c (and Figs. 2b in the main text) show that the supercurrent oscillations are less regular when $\theta \neq 90^\circ$, and display abrupt jumps. We hypothesize that these observations are related to the magnetization of the $NiPS_3$ layer. This interpretation is supported by the fact that the regularity and periodicity of SQUID oscillations are affected to a much lower extent when the magnetic field sweep direction is inverted for $B \parallel \hat{y}$, as shown in S9.

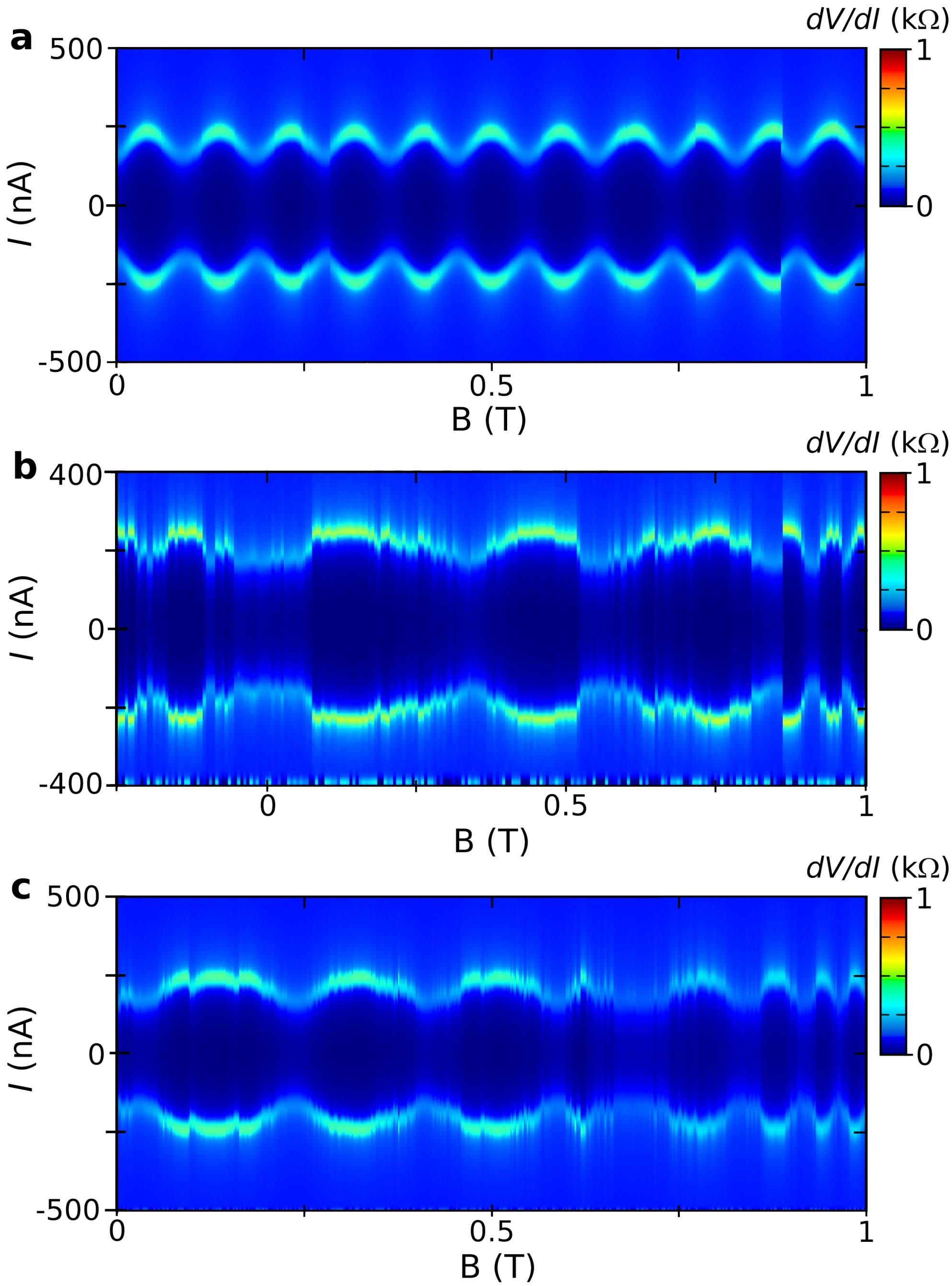

FIG. S8. $dV(I)/dI$ as a function of the magnetic field applied along $\theta =$ (a) 90°, (b) 130° and (c) -60°.

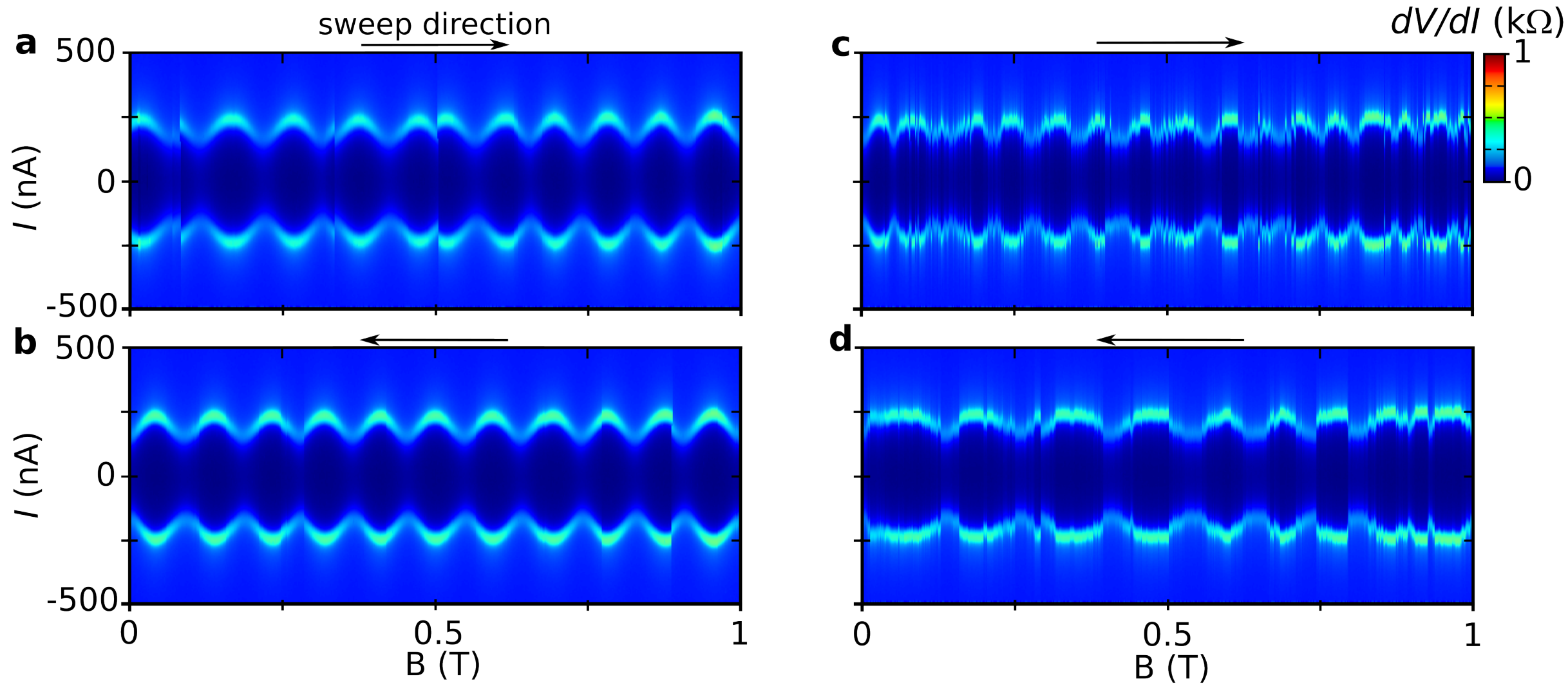

FIG. S9. Effect of the magnetic field sweep direction on the SQUID oscillations for (a), (b) $\theta = 90°$, i.e., $B \parallel \hat{y}$, and (c), (d) $\theta = 0°$, i.e., $B \parallel \hat{x}$. The arrow on top of each measurement represents the sweep direction.

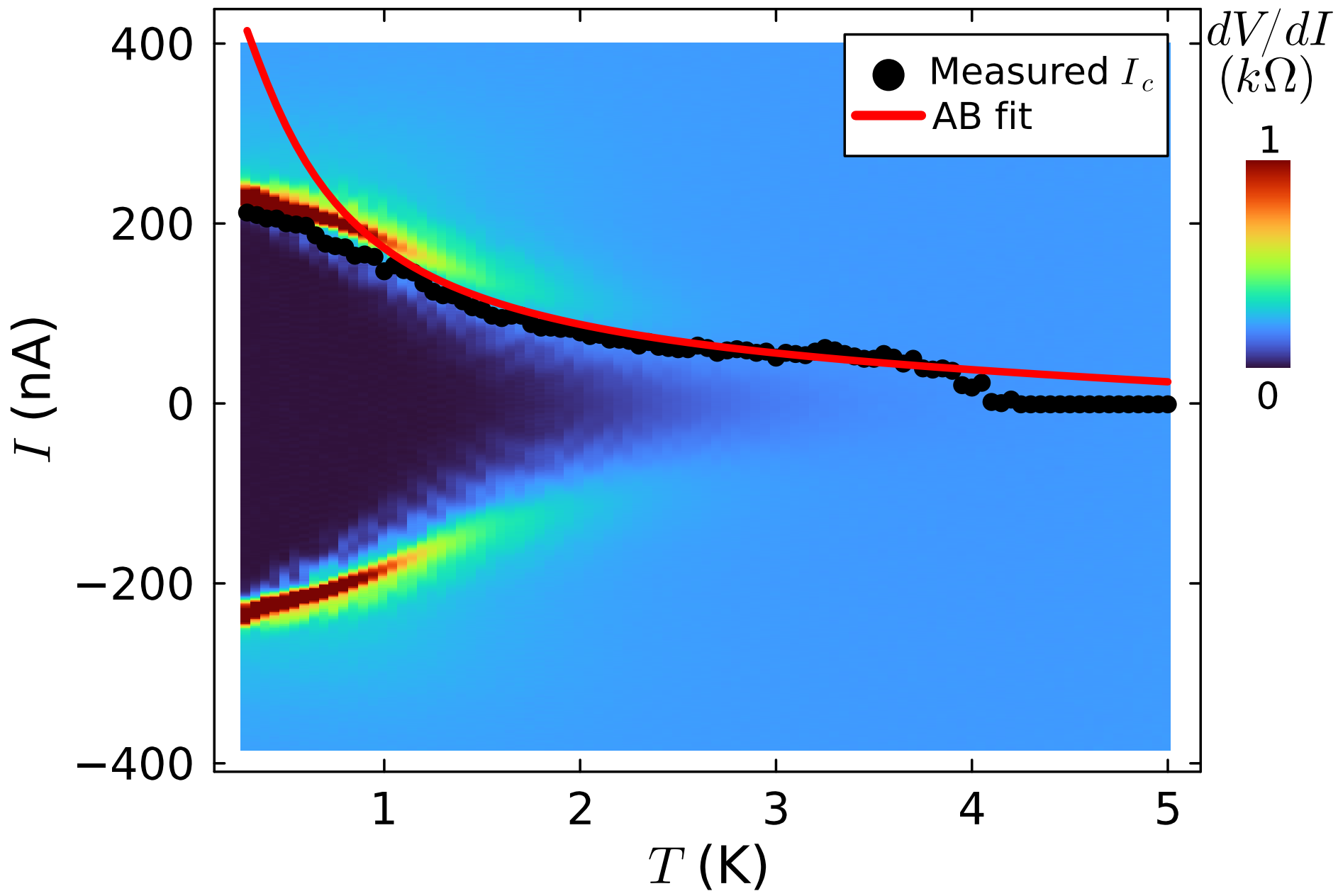

FIG. S10. Temperature dependence of the critical current. Differential resistance of the junction (color map) as in Fig. 1c of the main text. Fitting of the measured I_c values (black dots) using Ambegaokar-Baratoff formula (red line), Eq. (2), with parameters $\Delta/\Delta_{\mathrm{NbSe_2}} = 0.048$ and $T_c = 7.2$ K.

C. Temperature dependence of the critical current

Figure S10 shows the differential resistance of the junction, dV/dI, as a function of temperature (same as in Fig. 1c of the main text). The normal resistance, $R_N \approx 200\ \Omega$, is determined from the zero-bias value of dV/dI at $T = 5$ K. We then estimate from the data the critical current, $I_c(T)$, by setting a threshold corresponding to 95% of the differential resistance value in the normal state, namely,

$$\left.\frac{dV(I,T)}{dI}\right|_T = 0.95\left.\frac{dV(I=0,T)}{dI}\right|_{T=5K}. \tag{1}$$

Using this threshold, we obtain a set of experimentally measured $I_c(T)R_N$ points for fitting (black dots in Fig. S10).

As it is commonly done in the literature [4–8], we compare the critical current to the Ambegaokar-Baratoff formula,

$$I_c^{\mathrm{AB}} R_N = \frac{\pi\Delta(T)}{2e}\tanh\left(\frac{\Delta(T)}{2k_BT}\right), \tag{2}$$

with k_B being the Boltzmann constant, e the electron charge and where the temperature dependence of the gap is computed using the interpolating formula

$$\Delta(T) = \Delta_0 \tanh\Big(1.74\sqrt{T_c/T - 1}\Big), \tag{3}$$

for Δ_0 being the zero-temperature gap and T_c the critical temperature. The Ambegaokar-Baratoff formula is typically justified for insulating barriers [9].

The fitting parameters for the Ambegaokar-Baratoff formula are thus Δ_0 and T_c. In the fitting procedure we have imposed some constraints on the superconducting gap and critical temperature for $NbSe_2$ superconductors: The maximum allowed value for the gap is $\Delta_{\mathrm{NbSe_2}} = 1.26$ meV, based on bulk $NbSe_2$ as reported in [10]; while the maximum allowed critical temperature is $T_c^{\mathrm{NbSe_2}} = 7.2$ K [10]. This choice yields a ratio $\Delta_{\mathrm{NbSe_2}}/(k_B T_c^{\mathrm{NbSe_2}}) = 2.0$, consistent with BCS theory [11].

Using Eq. (2) with Δ_0 and T_c as fitting parameters with these constraints, we obtain the fit in Fig. S10 (red line) by maximizing the number of matching experimental points. Note that the fit works well for the middle range of temperatures, but fails at low temperatures and as the temperature approaches T_c.

To improve the fit of the $I_c(T)$ curve, we consider a quasi-one-dimensional Josephson channel with a degeneracy of g modes following the Furusaki-Tsukada formula [12]

$$I(\phi, T)R_N = \frac{\pi\Delta(T)}{2ge}\sin\phi\sqrt{\frac{1+Z^2}{\cos^2(\phi/2)+Z^2}}\tanh\left(\frac{\Delta(T)}{2k_BT}\sqrt{\frac{\cos^2(\phi/2)+Z^2}{1+Z^2}}\right), \tag{4}$$

with ϕ being the phase difference and where the parameter Z controls the junction's transparency. The Ambegaokar-Baratoff formula corresponds to the $Z \to \infty$ limit. We can use the normal-state version of Eq. (4) to identify the measured R_N as the resistance of a normal-state metallic contact with g transmitting channels, namely,

$$R_N^{-1} = g\frac{2e^2}{h}\frac{1}{1+Z^2}, \tag{5}$$

assuming that all channels are equivalent and have a transmission probability $1/(1+Z^2)$. The critical current is then obtained for the phase difference $\phi_{\max}$ that maximizes the Furusaki-Tsukada formula, Eq. (4), $I_c(T) = I(\phi_{\max}, T)$.

Figure S11 compares the multichannel Furusaki-Tsukada formula in the tunnel limit ($Z \gg 1$, blue line) to the single-channel Ambegaokar-Baratoff formula used in Fig. S10 (red line).

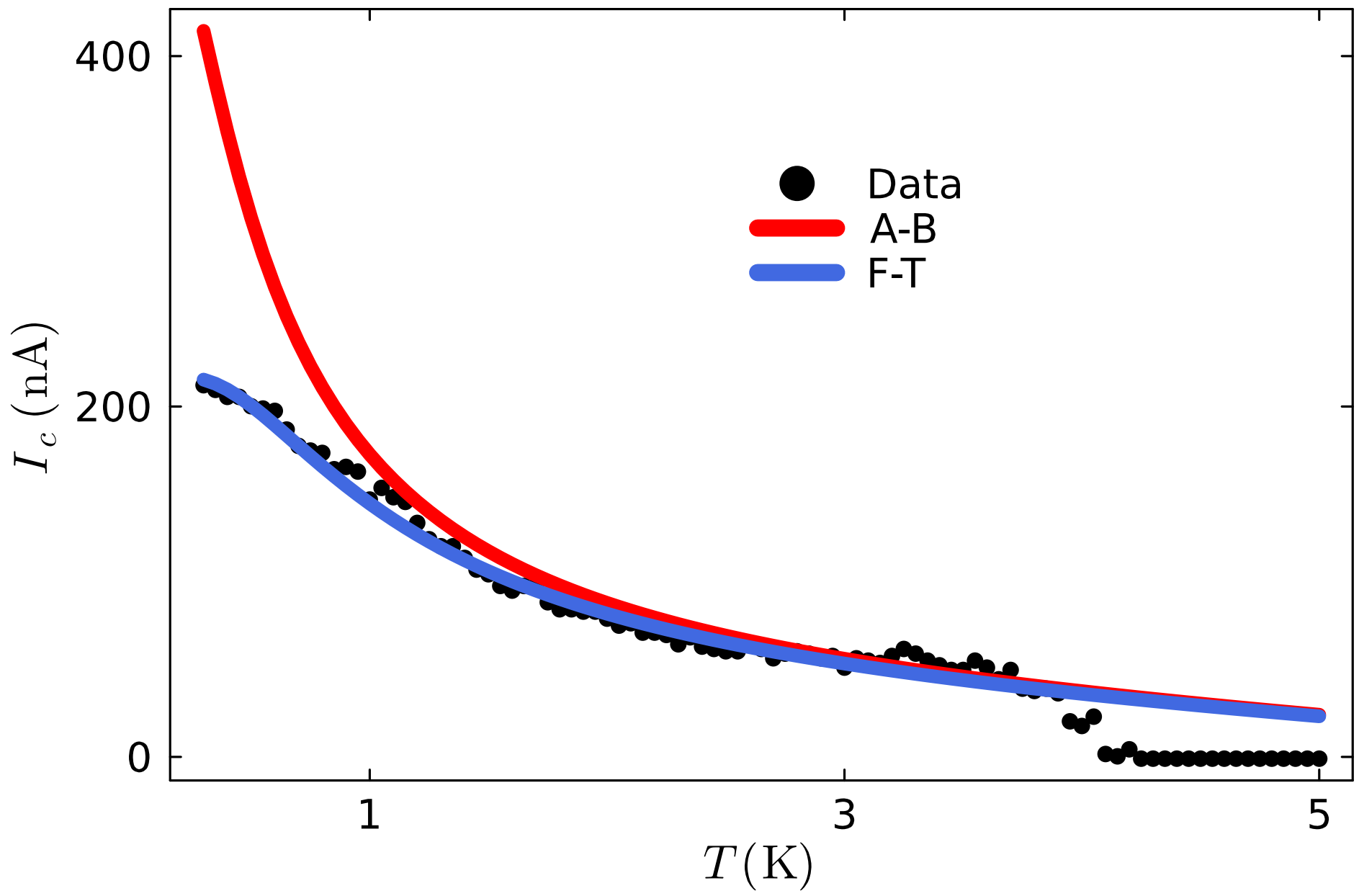

FIG. S11. Single-mode and multi-mode Ambegaokar-Baratoff formula. We compare the single-mode fit of Fig. S10 (red line, A-B) to a multi-mode fit using Eq. (4) with $g = 5$ modes and $Z \gg 1$ (blue line, F-T). The fitting parameters for the blue line are $\Delta = 0.11\Delta_{\mathrm{NbSe_2}}$ and $T_c = 7.2$ K.

Setting $g = 5$ allows us to fit the low temperature regime, but Eq. (4) still fails to reproduce the sharp drop in the critical current close to T_c.

Finally, we consider Eq. (4) with a finite barrier strength $Z \in [0, \infty)$. To match the critical voltage at zero temperature, the lowest measured voltage $V_c(T \simeq 0)$ is fitted to the expression

$$I_c R_N(0) = \frac{\pi\Delta(0)}{2ge} \sin\phi_{\max} \sqrt{\frac{1+Z^2}{\cos^2(\phi_{\max}/2) + Z^2}}. \tag{6}$$

We set the critical temperature T_c to the value in the literature ($T_c = 7.2$ K), and use the effective superconducting gap Δ and the barrier strength Z as fitting parameters. With these constraints, the model can be used to fit either the "head" (low temperature) or the "tail" (sharp drop around $T \sim 4$ K) of the experimental $I_c(T)$ curve, as shown in the left panel of Fig. S12. However, both regimes cannot be fitted simultaneously within this model. We can, instead, fit almost all experimental data points by considering two different contributions to the critical current, see purple line in Fig. S12(b). This result indicates that alternative transport mechanisms are taking place that are not considered by the model in Eq. (4). For example, the localized channels discussed in the main text.

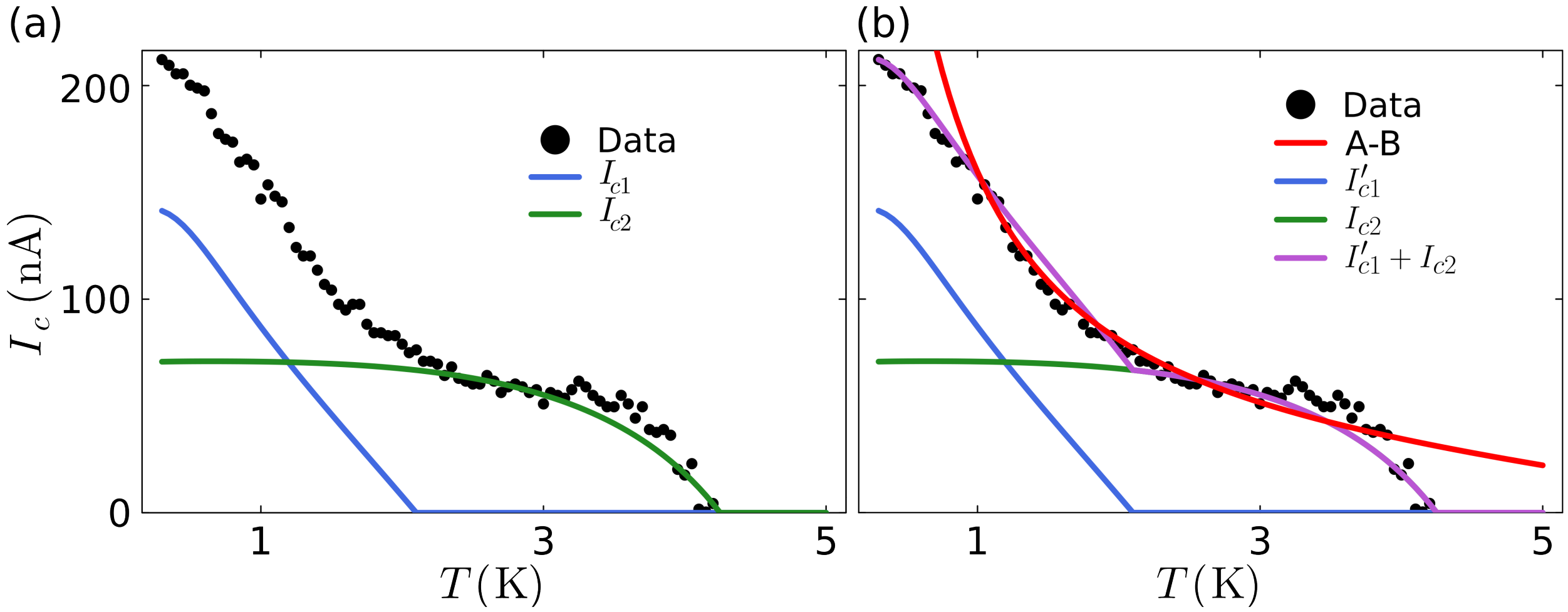

FIG. S12. Fitting of experimental data using two multi-mode currents. (a) The multi-mode Furusaki-Tsukada formula, Eq. (4), can be used to fit either the low temperature regime, I_{c1} (blue line), or the drop around $T \sim 4.2$ K, I_{c2} (green line), but not both. The fitting parameters used for I_{c1} are $\Delta_1 = 0.11\Delta_{\mathrm{NbSe_2}}$, $Z_1 = 1000$, $g_1 = 6$; and for I_{c2} we have $\Delta_2 = 1.0\Delta_{\mathrm{NbSe_2}}$, $Z_2 = 1000$, $g_2 = 140$. (b) A combination of two currents with different parameters, $I'_{c1} + I_{c2}$ (purple line), can fit all the features from the experimental data. I_{c2} is the same as in (a), while I'_{c1} has the same parameters as I_{c1} in (a) but with $T_c = 2.1$ K and $g = 8$. We show in red the Ambegaokar-Baratoff fit from Fig. S10 for comparison.

D. Magnetic field dependence of the critical current

The spatial distribution of supercurrent across a Josephson junction, $J(x)$, is connected to the modulation of the critical current with an applied magnetic field, $I_c(B)$ [13]. For example, assuming a sinusoidal current-phase relation we have [13]

$$J(x) = \frac{1}{2\pi}\int_{-\infty}^{\infty} d\beta\ I_c(\beta)\exp[i(\theta(\beta)-\beta x)], \quad \beta = 2\pi(2\lambda+t)\frac{B}{\Phi_0}, \tag{7}$$

where λ is the superconductor penetration length in the direction of the junction, t is the thickness of the barrier, and $\Phi_0 = h/(2e)$ is the flux quantum. The quantity $I_c(\beta)\mathrm{e}^{i\theta(\beta)}$, with both $I_c(\beta)$ and $\theta(\beta)$ real, and $I_c(\beta)$ corresponding to the experimental measurements, corresponds to the complex Fourier transform of the current density $J(x)$. Therefore, while $I_c(\beta)$ is known, the phase $\theta(\beta)$ must be determined under some assumptions.

Typically, if $\theta(\beta)$ has a minimum-phase property, it can be reconstructed assuming a nearly symmetric distribution for $J_c(x)$ [14, 15] or using a Hilbert transform [13]. In general, however, $\theta(\beta)$ may not be a minimum-phase function [16, 17]. Alternative approaches have been developed for these cases, including self-consistent schemes [18, 19] and maximum-entropy reconstructions using Monte Carlo simulated annealing [20, 21].

In the K06 sample that we focus on in the main text the current distribution can not be symmetric, since $I_c(B) \neq 0$ for all applied magnetic fields. However, for the right orientations of the magnetic field, $I_c(B)$ exhibits stable and periodic oscillations analogous to those of an asymmetric SQUID. We therefore propose a simple SQUID model that serves as a maximum-entropy solution for $J(x)$. We also assume a sinusoidal current-phase relation, which allows us to use Eq. (7), where we set $\lambda_\perp \approx 5$ nm [22] and estimate t to be at most 10 nm from our measurements. Any deviations from a sinusoidal current-phase relation would be reflected in the profile of $I_c(T)$. Note that non-sinusoidal current-phase relation does not imply low transparency; a small induced $\Delta/(k_BT)$ can also cause the superconducting phase to relax to $\phi^* = \pi/2$, independently of the junction transparency, as can be seen by taking the limit $\Delta/(2k_BT) \ll 1$ in Eq. (4).

In Fig. 2e of the main text we focused on the main Fourier contribution to the oscillations (that is, disregarding the shift due to the asymmetry of the channels) to compute the distance between the channels. We show in the top panels of Fig. S13 the same Fourier transform of Fig. 2e of the main text, together with the experimental measurements of $I_c(B)$. The

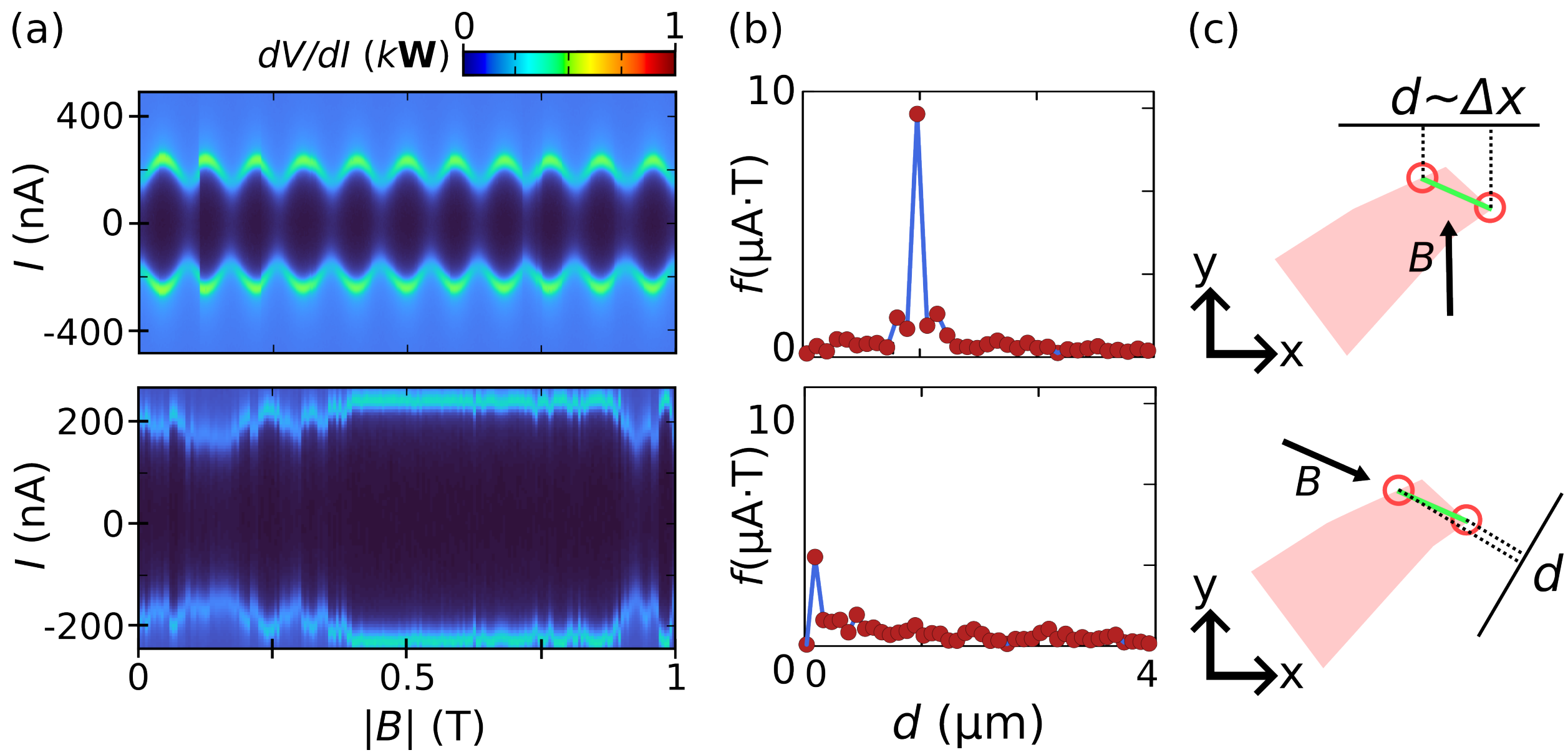

FIG. S13. Supercurrent interference pattern. (a) dV/dI as a function of the bias current and of an external magnetic field B applied at different orientations, as in Fig. 2b,c from the main text. (b) Main Fourier component of the critical current oscillations from the left panels, without the offset due to the asymmetry. (c) Magnetic field orientation with respect to the sample, together with the projection of the distance between channels along the direction perpendicular to B.

single peak of the Fourier transform, assuming a SQUID model, is thus consistent with the Josephson transport being dominated by two sets of localized channels separated a distance $d \sim 1\mu$m. This is confirmed when the oscillations disappear when we change the orientation of the magnetic field, see bottom panels of Fig. S13.

A simple SQUID model with sinusoidal current-phase relation is enough to interpret the experimental data as originated from two sets of localized channels across the junction. In the remainder of this section, we further analyze the Fourier transform of the measured $I_c(B)$ and refine our model to better estimate (i) the channel separation; (ii) the peak widths and peak current; and (iii) the angle yielding maximum SQUID oscillations from $dV(I = 0; B_x, B_y)/dI$.

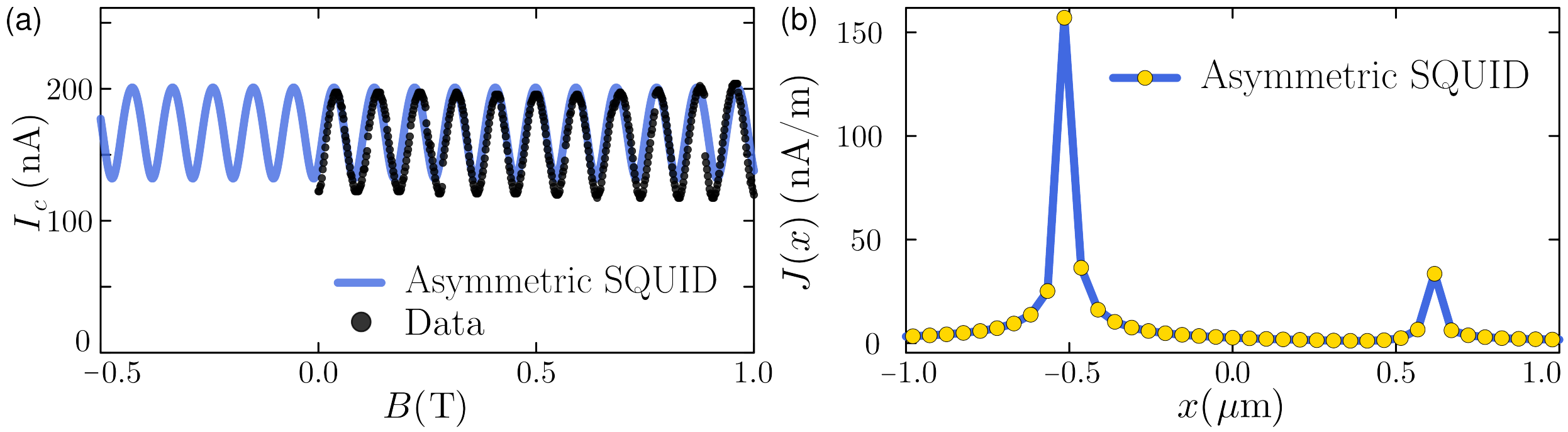

FIG. S14. Asymmetric SQUID model with no peak width. (a) Asymmetric SQUID model with $d = 1.24\ \mu$m (blue line) compared to the experimental data from sample K06 (black dots). (b) The Fourier transform yields a distance between channels of 1.24(5) μm.

1. *Zero-width asymmetric SQUID model*

To fit the experimental data we assume a SQUID model with two different current contributions, namely,

$$J_c(B) = A[\cos(\pi B d + \theta) + i \sin(\pi B d + \theta + \phi)], \tag{8}$$

so that $I_c(B) = |J_c(B)|$. Equation (8) is the Fourier transform of

$$J_c(x) = A\sqrt{2\pi}\left[\delta(x - d/2)e^{-i\theta}(1 + e^{-i\phi}) + \delta(x + d/2)e^{i\theta}(1 + e^{i\phi})\right], \tag{9}$$

that is, a setup with two zero-width (Dirac-delta) channels separated by a distance of d, whose amplitudes depend on a relative phase ϕ. The angle θ does not affect the channel amplitudes, only the position of the central lobe in the (B_x, B_y) plane.

We compare the asymmetric SQUID in Eq. (8) (blue line) to the experimental data from sample K06 (black dots) in Fig. S14(a) and check that the period and the small amplitude variation of the data are well reproduced. This way we obtain the channel asymmetry angle ϕ from the offset of $I_c(B)$ with respect to zero, and the oscillation amplitude A and phase shift θ from the data. Using the fitting parameters we estimate a distance between the peaks of 1.24(5) μm. This distance is compatible with corner modes in the transport region. In Fig. S14(b) we use the fitting parameters to compute Eq. (9). Note that the peaks in Fig. S14(b) feature a finite width due to Fourier uncertainty [17]. That is, the finite sampling of $I_c(B)$ in the range from 0T to 1T [yellow dots in Fig. S14(b)], result in an uncertainty of ~ 50 nm in the Fourier transform, comparable to the peak width.

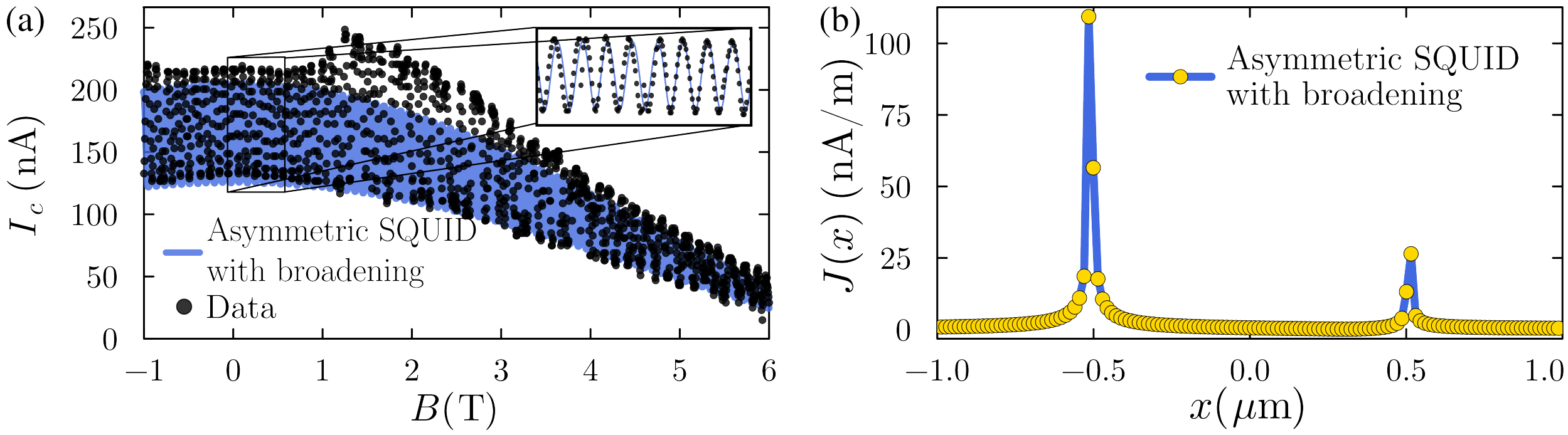

FIG. S15. Finite-width asymmetric SQUID model. (a) Data for sample K06 over a larger range of magnetic fields (black dots) compared Eq. (10) (blue line). The inset shows that the data periodicity between 0 and 0.5 T is captured for $d = 1.24$ μm, $\theta = 0.1\pi$, while the amplitude of the oscillations and the Fraunhofer-like decay are given by $\phi = 0.15\pi$, $\Omega = 0.044\pi$. (b) Fourier transform of Eq. (10) shows two peaks separated by a distance of $d = 1.24$ μm, with a broadening of ~ 40 nm.

2. *Finite-width asymmetric SQUID model*

To determine the width of the modes, we consider a second model which convolutes the sinusoidal dependence in Eq. (8) with a Fraunhofer pattern,

$$J_c'(B) = \mathrm{sinc}(\Omega B) J_c(B) = A\mathrm{sinc}(\Omega B)[\cos(2\pi Bd/2 + \theta) + i\sin(2\pi Bd/2 + \theta + \phi)]. \quad (10)$$

Here, the frequency Ω induces a Fraunhofer decay, so we expect $\Omega \gg d/2$. In real space, this corresponds to a small broadening. To analyze this, we fit the experimental data over a much larger integration window, from -1 to 6 T, corresponding to a sampling rate of 15 nm, see Fig. S15(a). The resulting peak broadening, defined as the width at which the amplitude decreases to 10% of its peak value, is approximately 40 nm, as shown in Fig. S15(b).

The peak width obtained is compatible with the sample dimensions, and would correspond to rather localized conduction channels.

3. *Relative angle between localized channels*

The differential resistance at zero current, $dV(I = 0)/dI$ (top panels of Fig. S16), shares the same periodicity as $I_c(B) = |J(B)|$, but does not feature the offset with respect to 0 that $I_c(B)$ displays, characteristic of the asymmetric SQUID pattern. We can thus estimate the position of the transport channels in the (x, y) plane where the magnetic field is applied

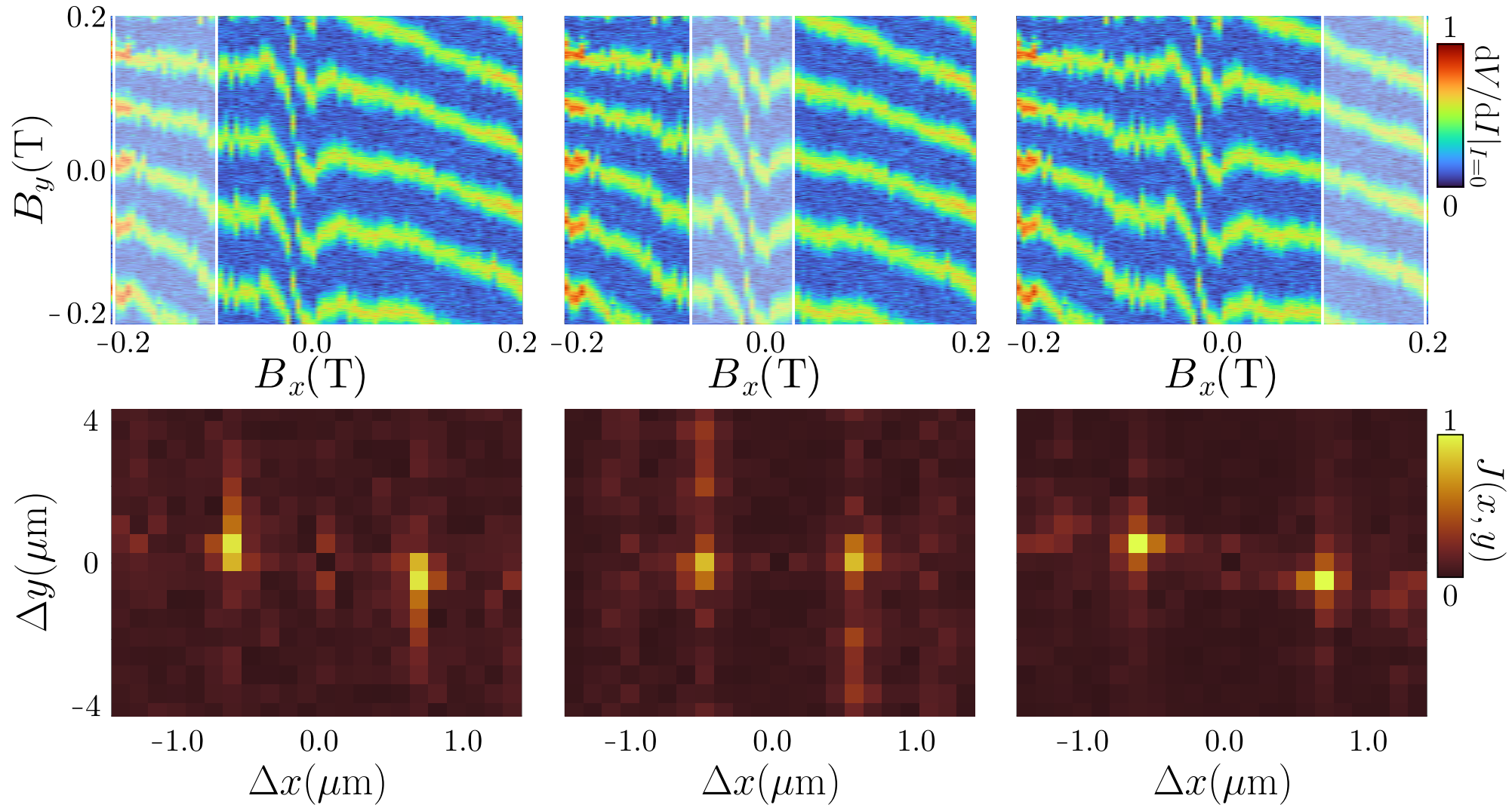

FIG. S16. Two-dimensional Fourier transform of $I_c(B_x, B_y)$. From left to right, different ranges of B_x are considered. Their corresponding 2D Fourier transforms are below, with maximum peak intensity shown in brighter colors.

assuming a symmetric SQUID model with sinusoidal current-phase relation. Using this model, the two-dimensional Fourier transform of the oscillations of the differential resistance data in (B_x, B_y) space reveals the relative distance and angle between conducting channels. As a result, a two-dimensional map of the Fourier transform creates an image of the channel positions, see bottom panels of Fig. S16.

As the top panels of Fig. S16 show, the data periodicity is not uniform with the magnetic field. Upon varying B_x, the periodic pattern changes between positive and negative values, and features a phase slip around zero. The position of the localized channels thus changes with the field. To better represent this dependence of the distance between channels with B_x, we perform a two-dimensional Fourier transform of different regions of the magnetic field map, indicated by shaded areas in the top panels of Fig. S16. As a result, we obtain $J(x, y)$, which generalizes Eq. (7) extending it to the (x, y) plane. In all cases, including the region around $B_x \sim 0$ T featuring a phase slip, the computed $J(x, y)$ features two localized peaks mostly separated along the x-direction. The mean distance between peaks in the direction perpendicular to the oscillations is within $1-2$ microns, with a maximum distance of 1.7 μm. We plot the distance as a function of B_x in the left panel of Fig. S17 to directly compare the different regions. The peak intensity, which indicates the resolution of the

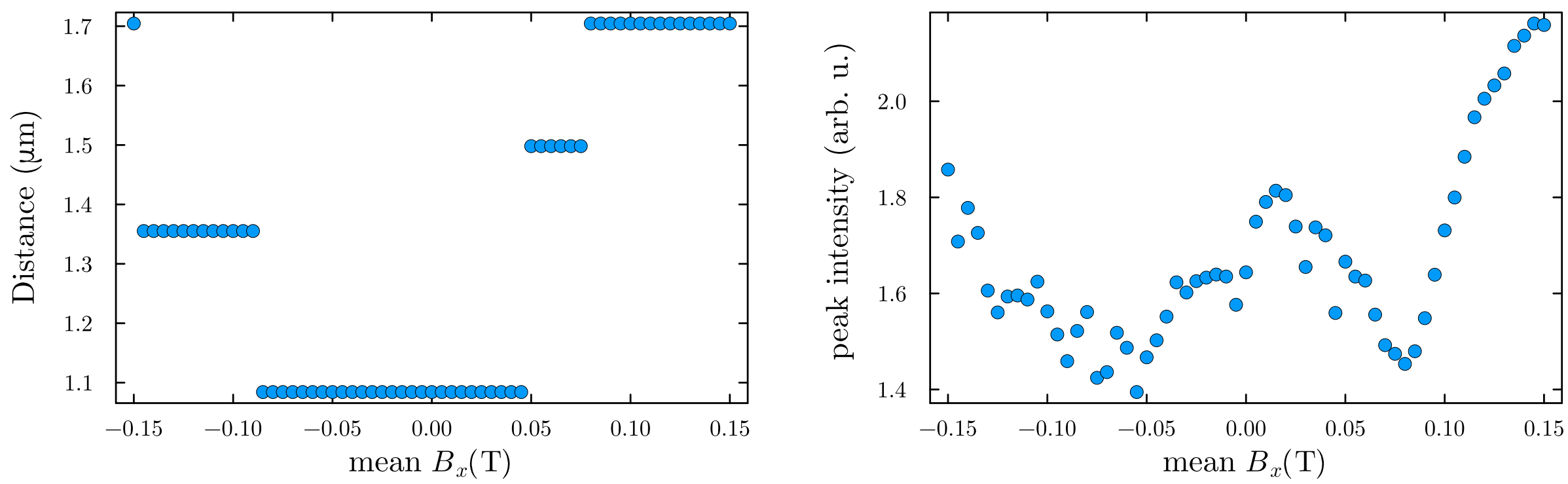

FIG. S17. Peak distance (left) and intensity (right) estimated from the two-dimensional Fourier transform. (a) Distance $|d| = \sqrt{\Delta x^2 + \Delta y^2}$ as a function of mean B_x for each integration window shown in Fig. S16. (b) Peak Fourier component (arbitrary units) as a function of mean B_x.

Fourier transform, is shown in the right panel of Fig. S17. The central region for $B_x \sim 0$ T features the smallest distance, of around one micron, with an average resolution. The largest distance corresponds to the regular area at finite and positive B_x, which also features the highest peak intensity.

S5. MICROSCOPIC MODEL

We simulate the interface between the superconductor $NbSe_2$ and the antiferromagnet $NiPS_3$ using a square lattice tight-binding model of dimensions $N_x a$ and $N_y a$, with a being the lattice parameter. The tight-binding Hamiltonian reads

$$H = H_{\rm SC} + H_{\rm AF}, \tag{11}$$

with $H_{\rm SC}$ corresponding to the superconducting $NbSe_2$ and $H_{\rm AF}$ representing the magnetic effect induced by the $NiPS_3$. Specifically,

$$H_{\rm SC} = -\sum_{\mathbf{r},\sigma} \mu(\mathbf{r}) c^\dagger_{\mathbf{r},\sigma} c_{\mathbf{r},\sigma} - t \sum_{\langle \mathbf{r},\mathbf{r}'\rangle,\sigma} c^\dagger_{\mathbf{r},\sigma} c_{\mathbf{r}',\sigma} \sum_{\mathbf{r}\sigma} + \Delta(\mathbf{r}) c^\dagger_{\mathbf{r},\sigma} c^\dagger_{\mathbf{r},\bar{\sigma}} + \text{H.c.}, \tag{12a}$$

$$H_{\rm AF} = \sum_{\mathbf{r},\sigma,\sigma'} \mathbf{M}_\mathbf{r} \cdot \boldsymbol{\sigma}_{\sigma\sigma'} c^\dagger_{\mathbf{r},\sigma} c_{\mathbf{r},\sigma'} + \text{H.c.} \tag{12b}$$

Here, $c^\dagger_{\mathbf{r},\sigma}$ and $c_{\mathbf{r},\bar{\sigma}}$ respectively create and annihilate electrons with spin $\sigma = \uparrow, \downarrow$ ($\bar{\sigma} = \downarrow, \uparrow$) on the square lattice site $\mathbf{r} = (x, z)$, $\mu(\mathbf{r})$ is the chemical potential, t is the nearest-neighbor hopping amplitude, Δ is the homogeneous superconducting pairing potential, $\mathbf{M}_\mathbf{r}$ is an on-site magnetic term, and $\boldsymbol{\sigma} = (\hat{\sigma}_1, \hat{\sigma}_2, \hat{\sigma}_3)$, with $\hat{\sigma}_{1,2,3}$ being the spin Pauli matrices. The total system size is $N_x \times N_y$, but we are interested in the interface region where the SC and AF coexist, a square of size $l_x \times l_y$ with $l_{x,y} < N_{x,y} a$, where we consider $\Delta(\mathbf{r}) \neq 0$ and $\mu \ll 4t$. Outside this reduced region we have $\mu(\mathbf{r}) \gtrsim 4t$ and $\Delta(\mathbf{r}) = 0$, while $\mathbf{M}_\mathbf{r}$ is finite everywhere.

To represent the magnetic texture of $NiPS_3$ in the square lattice we consider the crystallographic b axis where the spins align antiferromagnetically to coincide with the x direction, compare Fig. 1a and Fig. 3a of the main text. Along this direction, the spins follow a sequence of two sites with $\uparrow$ spins, a neutral site, two $\downarrow$ spins, and another neutral site, namely,

$$(M_{x+1,y}, M_{x+2,y}, ..., M_{x+6,y}) = (m, m, 0, -m, -m, 0)\hat{\sigma}_3.$$

Here, m represents the on-site magnetization strength. The pattern is repeated in the x direction while maintaining the same spin orientation for all sites in the y direction. First, we choose $l_x = (6p-1)a$, with p a positive integer, so that the system is globally compensated, i.e., starts with a $+m$ site and ends with a $-m$ one. As a result, the Hamiltonian maintains a symmetry that combines time-reversal and inversion symmetry along the x-direction. Taking also $l_y = l_x$, we make sure that the system is Kramers-degenerate. We can then tilt the square region $l_x \times l_y$ by an angle θ to explore different possibilities.

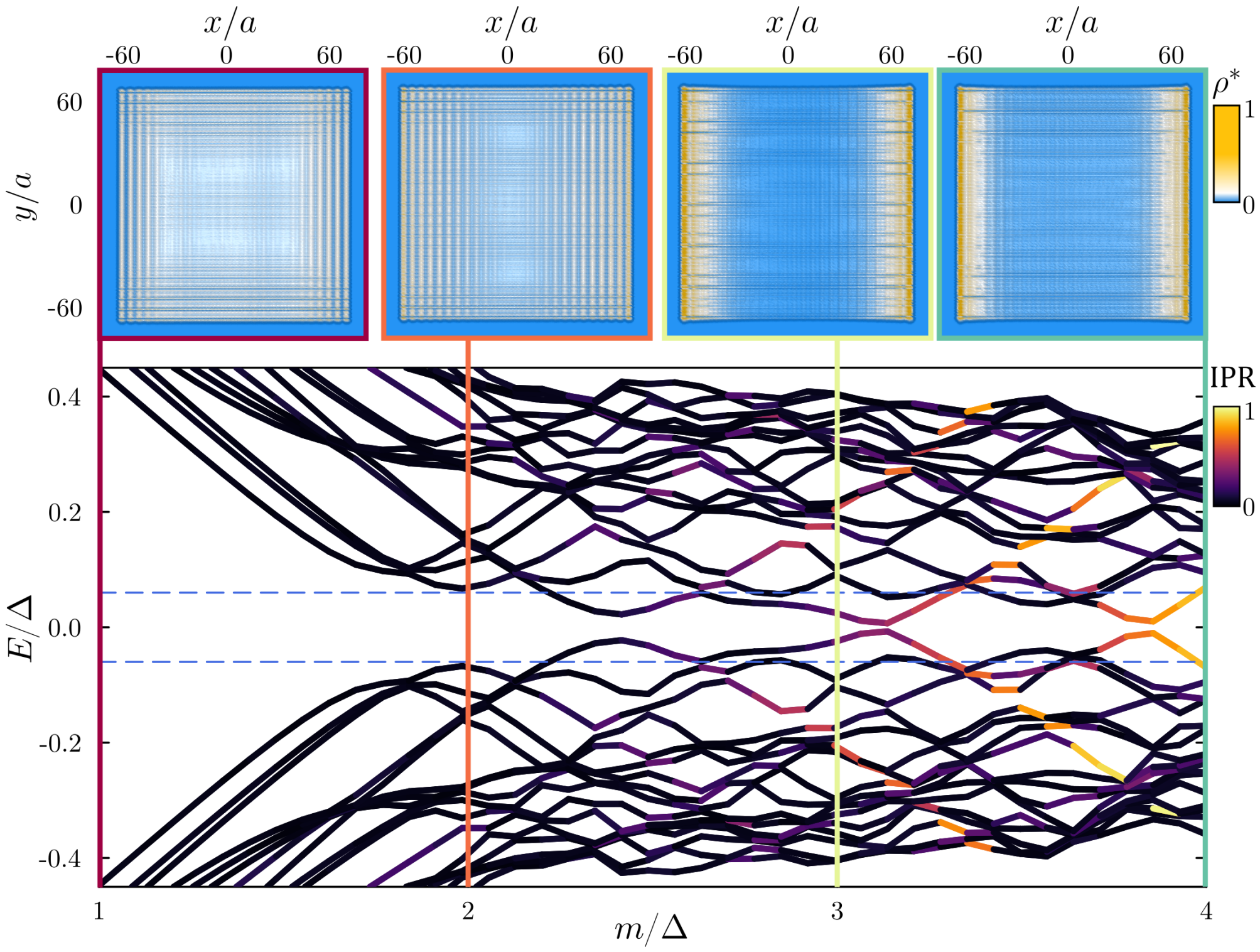

FIG. S18. Magnetically-induced subgap states. Low-energy spectrum of the Hamiltonian in Eq. 11 as a function of the magnetization strength m. Each energy band is colored with the inverse participation ratio, IPR, of its corresponding eigenvector. The top panels show the weighted density of states, Eq. (13), for $m/\Delta = 1, 2, 3$, and 4, as indicated by the colored lines. The superconducting region has size $l_x = l_y = 120a$ and $\mu = -2.2t$ and $\Delta = 0.1t$. This region is embedded in a magnetic insulator of size $N_x = N_y = 144$, where $\Delta = 0$ and $\mu = -4.1t$.

The combination of superconductivity and the spin symmetry breaking at the internal edges of the superconducting region ($l_{x,y}$) favors the formation of low-energy subgap states. We plot the low-energy spectrum as a function of the magnetization strength m in Fig. S18 and observe how these states can reach very low energies and localize in the edges of the system when $m \gtrsim \Delta$ (top panels with $m/\Delta = 1, 2, 3, 4$). We use the inverse participation ratio, $\mathrm{IPR} = \sum_{x,y} |\psi(x, y)|^4$, to track the state localization.

The low-energy states require the simultaneous presence of superconducting pairing, Δ, and magnetization, m. The superconducting region, of area $l_x \times l_y \leq N_x a \times N_y a$, defines some internal boundaries where spins can align antiferromagnetically or ferromagnetically, see green squares in Fig. S19. As we demonstrated in the main text, see Fig. 3, the low-

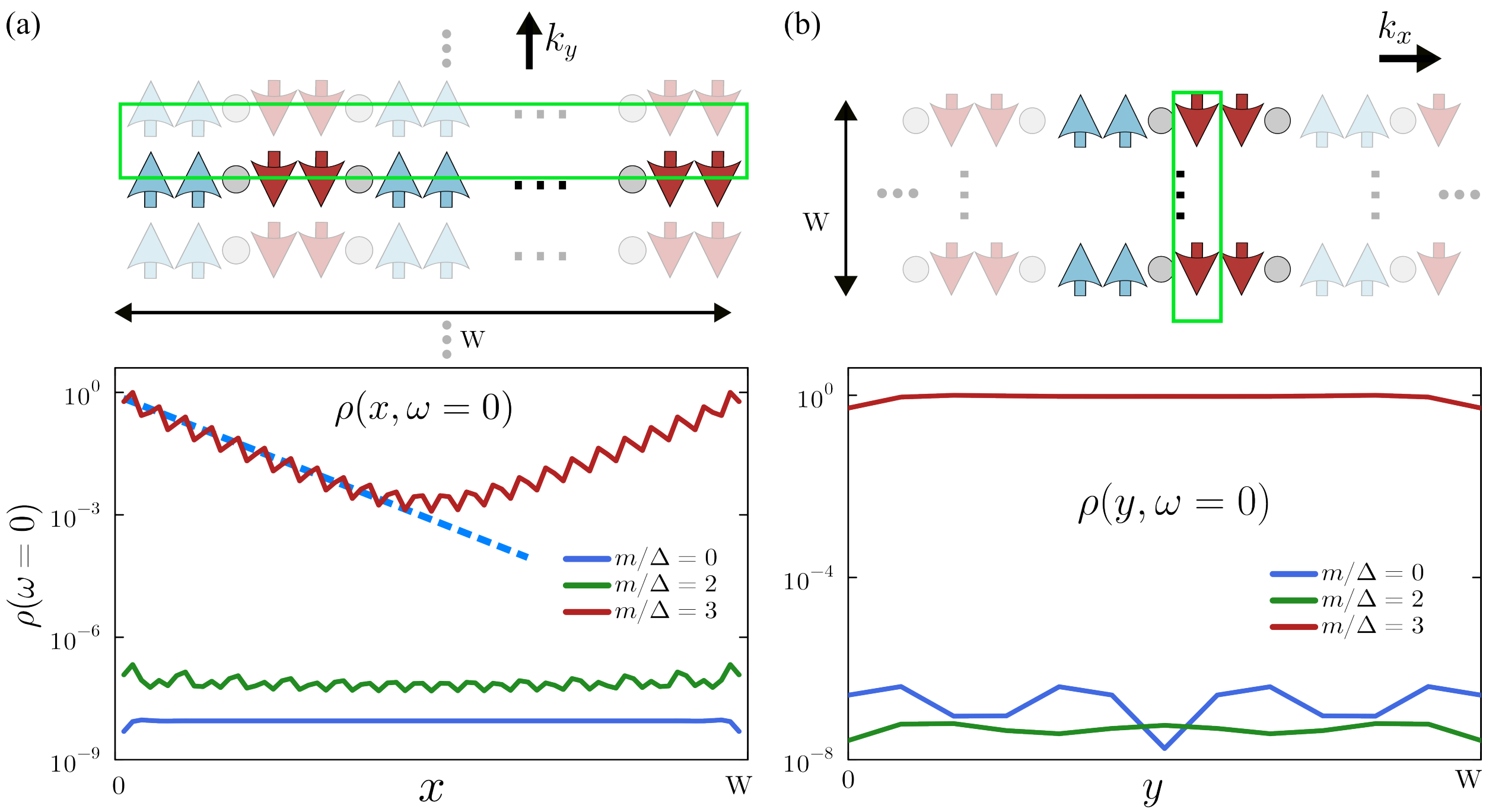

FIG. S19. Localization of low-energy states. Density of states at $\omega = 0$ as a function of the system width in semi-logarithmic plot, applying periodic boundary conditions (a) along the y direction to obtain antiferromagnetic edges on the x axis; or (b) along the x direction so that ferromagnetic edges form on the y axis. The blue dashed line in (a) indicates the exponential decay of the zero-energy density of states. The top panels show a sketch of how the periodic boundary conditions are applied in each case, and the type of edges formed, with the green square indicating the respective unit cells. Parameters: $\mu/t = 3.75$, $\Delta/t = 0.2$, $m/\Delta = 3$.

energy states localize along the ferromagnetic boundaries. To study the spatial distribution of the low-energy states in more detail, we consider a configuration with a square superconducting region ($l_x = l_y$) oriented parallel to the larger magnetic region ($\theta = 0$). Since we aligned the $NiPS_3$ antiferromagnetic texture with the x-axis, see Eq. (12), that direction displays antiparallel spins while the spins along the y-direction are parallel. Consequently, by imposing periodic boundary conditions along either the (a) y- or the (b) x-directions, we build systems infinite in one direction but finite in the other direction, where boundaries are (a) ferromagnetic or (b) antiferromagnetic. For a large enough magnetic moment, $m \gtrsim \Delta$, we observe that the density of states at zero excited energy, $\rho(\omega = 0)$, displays exponential localization at the ferromagnetic edges of the sample, Fig. S19(a), while it remains delocalized for the antiferromagnetic edges, Fig. S19(b).

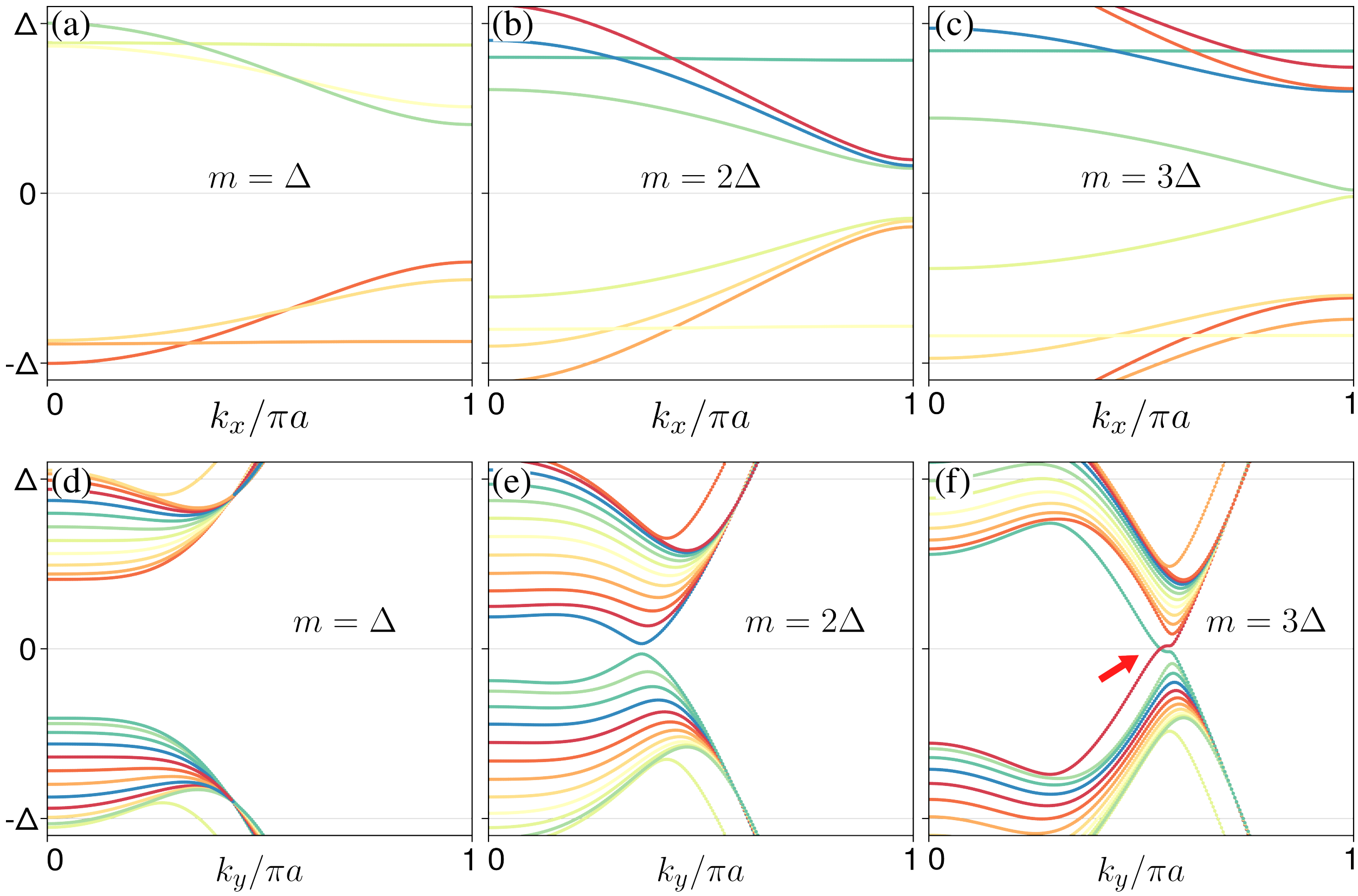

FIG. S20. Energy bands for the ribbon geometries. (a-c) Band dispersion for the ribbon with antiferromagnetic edges (periodic boundary conditions applied along the x direction). (d-f) Band dispersion for ribbons with uncompensated borders (periodic boundary conditions along y direction)). The red arrow in (f) marks the zero-energy crossing corresponding to the edge modes of the ferromagnetic boundaries. Parameters: $\mu/t = 3.75$, $\Delta/t = 0.2$.

The spectrum is Kramers degenerate since we are not including any spin-orbit coupling and we only consider systems that keep the combined time-reversal and inversion symmetries. For antiferromagnetic edges we observe a small spin splitting since the superconducting coherence length, $\xi_S \approx 2t/(\pi\Delta) \approx 6a$, is comparable to the magnetic length (spanning six sites). However, there is no band inversion and, therefore, only trivial topology, see Fig. S20(a-c). For ferromagnetic edges we do observe band inversion, Fig. S20(d-f), corresponding to a double fermion parity change owing to Kramers degeneracy. Consequently, this case also displays trivial topology, even though states localize at zero energy.

So far we only considered localization of states on a superconductor-antiferromagnet (SC-AF) planar interface. In the main text we hypothesize that transport across the junction is

facilitated by an overlap between states localized at different SC-AF interfaces. Assuming a symmetric junction, the transmission through localized states is inversely proportional to the square of the energy: $T = 4\Gamma^2/(E-\epsilon_F)^2$, with Γ being the symmetric coupling to a localized state with energy E over the Fermi energy ϵ_F. Consequently, the closer the localized states are to the Fermi energy, the more likely they are to open transport channels. In the main text, see Fig. 3, we defined a weighted density of states

$$\rho^*(x, y) = \sum_i |\psi_i(x, y)|^2/(\epsilon_i - \epsilon_F)^2, \tag{13}$$

which favors states with energies closer to the Fermi energy to better identify the localized states that are more likely to open transport channels between interfaces.

---

[1] C.-T. Kuo, M. Neumann, K. Balamurugan, H. J. Park, S. Kang, H. W. Shiu, J. H. Kang, B. H. Hong, M. Han, T. W. Noh, and J.-G. Park, Scientific Reports **6**, 20904 (2016).

[2] H. Lu, W. Wang, Y. Liu, Y. Li, Y. Li, Y. Li, Y. Li, Y. Li, Y. Li, and Y. Li, Applied Surface Science **504**, 144405 (2020).

[3] F. Fernandez-Cañizares, G. Sanchez-Santolino and collaborators, *private communication*.

[4] N. Yabuki, R. Moriya, M. Arai, Y. Sata, S. Morikawa, S. Masubuchi, and T. Machida, Nat. Commun. **7**, 1 (2016).

[5] L. S. Farrar, A. Nevill, Z. J. Lim, G. Balakrishnan, S. Dale, and S. J. Bending, Nano Letters **21**, 6725 (2021), pMID: 34428907, https://doi.org/10.1021/acs.nanolett.1c00152.

[6] L. Ai, E. Zhang, J. Yang, X. Xie, Y. Yang, Z. Jia, Y. Zhang, S. Liu, Z. Li, P. Leng, X. Cao, X. Sun, T. Zhang, X. Kou, Z. Han, F. Xiu, and S. Dong, Nat. Commun. **12**, 1 (2021).

[7] S. Chen, W. Tian, Z. Xu, P. Zhang, H. Du, Z. Wei, D. Li, Y. Lv, H. Sun, Y.-L. Wang, D. Koelle, R. Kleiner, H. Wang, and P. Wu, Phys. Rev. B **104**, 214512 (2021).

[8] C. Zhao, X. Yi, Q. Chen, C. Yan, and S. Wang, J. Phys. Chem. Lett. **13**, 10811 (2022).

[9] Y. Ota, N. Nakai, H. Nakamura, M. Machida, D. Inotani, Y. Ohashi, T. Koyama, and H. Matsumoto, Phys. Rev. B **81**, 214511 (2010).

[10] T. Dvir, F. Massee, L. Attias, M. Khodas, M. Aprili, C. H. L. Quay, and H. Steinberg, Nat. Commun. **9**, 1 (2018).

[11] N. K. Kuzmenko, Phys. C **576**, 1353709 (2020).

[12] S. Kashiwaya and Y. Tanaka, Rep. Prog. Phys. **63**, 1641 (2000).

[13] R. C. Dynes and T. A. Fulton, Phys. Rev. B **3**, 3015 (1971).

[14] S. Hart, H. Ren, T. Wagner, P. Leubner, M. Mühlbauer, C. Brüne, H. Buhmann, L. W. Molenkamp, and A. Yacoby, Nat. Phys. **10**, 638 (2014).

[15] R. Fermin, D. van Dinter, M. Hubert, B. Woltjes, M. Silaev, J. Aarts, and K. Lahabi, Nano Lett. **22**, 2209 (2022).

[16] H. H. Zappe, Phys. Rev. B **11**, 2535 (1975).

[17] A. Papoulis, The Fourier Integral and Its Applications (McGraw-Hill, New York, 1962).

[18] O. Nesher and E. N. Ribak, Appl. Phys. Lett. **71**, 1249 (1997).

[19] H.-Y. Hui, A. M. Lobos, J. D. Sau, and S. Das Sarma, Phys. Rev. B **90**, 224517 (2014).

[20] S. Ghatak, O. Breunig, F. Yang, Z. Wang, A. A. Taskin, and Y. Ando, Nano Lett. **18**, 5124 (2018).

[21] A. Zalic, T. Taniguchi, K. Watanabe, S. Gazit, and H. Steinberg, Nano Lett. **23**, 6102 (2023).

[22] H. Wu, Y. Wang, Y. Xu, P. K. Sivakumar, C. Pasco, U. Filippozzi, S. S. P. Parkin, Y.-J. Zeng, T. McQueen, and M. N. Ali, Nature **604**, 653 (2022).
